# *H*-linear magnetoresistance in NbSe_2_ due to impeded cyclotron motion

**DOI:** 10.1126/sciadv.aea6029

**Published:** 2026-04-29

**Authors:** Arwin Kool, Davide Pizzirani, Paul Tinnemans, Steffen Wiedmann, Felix Flicker, Jasper van Wezel, Nigel E. Hussey, Roemer D. H. Hinlopen

**Affiliations:** ^1^ High Field Magnet Laboratory (HFML-FELIX), Radboud University, Nijmegen, Netherlands.; ^2^ Institute for Molecules and Materials, Radboud University, Nijmegen, Netherlands.; ^3^ H. H. Wills Physics Laboratory, University of Bristol, Bristol, UK.; ^4^ Institute for Theoretical Physics, University of Amsterdam, Amsterdam, Netherlands.

## Abstract

Linear magnetoresistance (LMR) is a widespread phenomenon observed in a host of quantum materials ranging from semiconductor nanostructures to quantum critical and strange metals. While multiple scenarios to explain LMR have been proposed, a complete understanding of the phenomenon remains elusive. It is highly likely that the origin of LMR depends on the specific electronic state. Here, we report a study of the impact of disorder on the form of the magnetoresistance of the prototypical charge-density-wave (CDW) compound 2*H*-NbSe_2_. The magnetoresistance is shown to exhibit strong qualitative and quantitative agreement with Boltzmann transport analysis incorporating impeded cyclotron motion (ICM). We identify the source of ICM in 2*H*-NbSe_2_ as strong scattering sinks where the CDW order connects the high-temperature Fermi cylinders. Such unusual “hotspots” provide an explanation for the observed LMR as well as for the long-unexplained absence of quantum oscillations inside the charge-ordered state in 2*H*-NbSe_2_. These findings provide strong evidence that ICM generates LMR in certain correlated metals.

## Teaser

Linear magnetoresistance in NbSe_2_ is shown to be linked to impeded cyclotron motion induced by the low-temperature charge order.

## INTRODUCTION

Linear-in-field magnetoresistance (LMR) contrasts markedly with the usual quadratic-to-saturating shape of the MR found in conventional metals. This, coupled with its remarkable robustness (extending over an anomalously broad field range) and its realization in a host of different quantum materials, from semiconductors (*1*, *2*) and Dirac semimetals (*3*) to quantum critical metals (*4*–*6*) and doped Mott insulators (*7*–*9*), has led to sustained interest in the LMR phenomenon for well over half a century. Reflecting the breadth of material classes in which LMR has been observed, multiple proposals have been put forward for its origin. The first category of proposals centers on materials with a low or vanishing carrier density. In GaAs quantum wells, for example, carrier density fluctuations result in a nonsaturating LMR up to high magnetic fields (*1*, *2*). For more strongly disordered semiconductors, LMR can arise from the formation of an effective random resistor network (*10*, *11*), while LMR is also expected at high magnetic field strengths near the quantum limit, where only a few Landau levels remain occupied (*12*). For metals with a high carrier density, LMR can arise, albeit over a limited field range, from sharp corners on the Fermi surface (FS) (*13*–*15*).

The manifestation of LMR in correlated metals with relatively simple, large, cylindrical FS geometries, however, suggests an alternative origin possibly linked to the emergence of novel electronic states. In iron-based superconductors tuned to their quantum critical point, for example, a peculiar form of nonsaturating *H*^2^- to *H*-linear MR—with a *T*-independent slope—has been reported (*4*–*6*). The MR in these systems also exhibits a novel form of (non-Kohler) scaling in which magnetic field and temperature (rather than the zero-field resistivity) appear in quadrature (*4*). LMR with similar scaling properties has also been observed in the high-*T*_c_ cuprates, both close to the end of the pseudogap regime (*7*) and beyond (*8*, *9*). In the latter, the *T*-independent slope of the LMR is found to scale directly with both *T*_c_ and the coefficient of the low-*T T*-linear resistivity (*16*), suggesting a direct link to strange metallicity and high-*T*_c_ superconductivity.

The simplicity and robustness of the LMR found in the iron-based and cuprate superconductors have motivated a number of tailored theoretical proposals, including real-space binary distributions (*17*), real-space patches (*18*), carrier density fluctuations (*19*), and strong scattering sinks (*20*–*22*) among others. Proposals that incorporate scattering “hotspots” or any other obstruction to cyclotron motion on the FS can be generalized through the concept of impeded cyclotron motion (ICM) (*21*). In this picture, the orbital motion of carriers around the FS is truncated at specific loci, for example, through gapping, heavy effective masses, or intense scattering. These loci themselves are irrelevant for the resistivity of the material in the absence of a magnetic field due to shorting by the rest of the FS (which we will refer to as cold). With field applied, however, cyclotron motion of the cold carriers is impeded, resulting in a characteristic crossover from *H*^2^- to *H*-linear MR with a slope that is *T* independent (*21*). The universality of LMR in correlated metals is then explained by the wide range of possible sources of the impedances that might occur, including hotspot scattering off antiferromagnetic spin fluctuations (*20*, *23*), van Hove singularities (*24*), static domains of glassy density-wave order (*22*), Fermi arcs in underdoped cuprates (*25*), or partial FS incoherence (*9*). Hence, identifying materials where ICM can account for the form and magnitude of the LMR as well as the range in tuning parameters where LMR is observed would provide definitive evidence of a dominant (and strongly *k*-dependent) interaction.

**Fig. 1.**
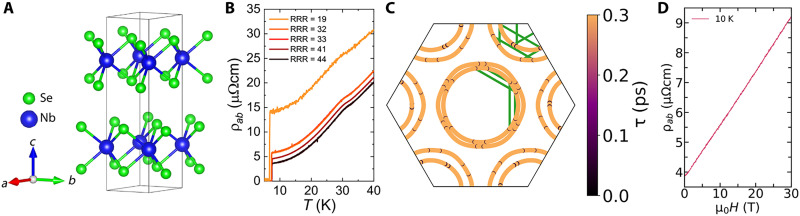
Lattice, resistivity versus temperature, and simulation of the FS of bulk NbSe_2_ in the CDW state. (**A**) Crystal structure of bulk 2*H*-NbSe_2_. The crystal structure consists of two weakly coupled, hexagonal Se-Nb-Se layers. (**B**) In-plane resistivity ρ*_ab_*(*T*) for five samples of NbSe_2_ of varying quality as indicated by the factor-of-four variation in the residual resistivity ρ_0_. The superconducting and CDW transition temperatures decrease monotonically with increasing ρ_0_ [decreasing residual resistivity ratio or RRR, consistent with previous disorder studies (*37*, *38*)]. (**C**) The tight-binding FS of bulk NbSe_2_ previously determined via ARPES (*35*). Unless mentioned otherwise, we ignore the small pancake-shaped pocket centered around the Γ-point. Instead of a FS reconstruction, impedances (black) are shown where the FS is connected intrapocket by the CDW $\vec{Q}$-vector (examples in green), while cold charge is colored orange. (**D**) Exemplary LMR measured on the sample with RRR = 44 at *T* = 10 K.

In this context, the prototypical charge-density-wave (CDW) compound 2*H*-NbSe_2_ (henceforth abbreviated to NbSe_2_) can be considered a viable candidate for the realization of ICM (*21*). Among the dichalcogenides, NbSe_2_ has a relatively low CDW transition temperature of *T*_CDW_ = 33 K and a high superconducting transition temperature of *T*_c_ = 7.2 K. As shown in Fig. 1A, NbSe_2_ is composed of Nb slabs sandwiched between Se atoms. LMR was first reported in NbSe_2_ more than 50 years ago (*26*) and ascribed to magnetic breakdown through the gaps opened by the CDW (*27*). Such behavior, however, is known to result in MR saturation rather than LMR (*28*–*30*). The lack of quantum oscillations (QOs) from all pockets which participate in the CDW order also remains notable and unexplained (*31*, *32*). However, if the CDW order manifests with a large imaginary self-energy for any reason (e.g., low single-particle lifetimes for phonon-dressed quasiparticles), then the resulting impedances to cyclotron motion could explain both a lack of QOs on the participating pockets and the emergence of LMR which disappears with increasing temperature or pressure in tandem with the CDW phase (*33*).

To test the applicability of ICM to NbSe_2_, we have carried out a magnetotransport study of bulk NbSe_2_ over an unprecedented field range as a function of temperature and disorder. The LMR is found to disappear upon approach to the CDW transition temperature from below and, to within our experimental accuracy, follows Kohler scaling up to this breakdown temperature. Recall that Kohler scaling is a single-parameter scaling of the magnetoresistance with ω_c_τ (*34*) that is expected semiclassically if the MR changes solely due to a *T*-dependent scattering rate. Kohler scaling is obeyed in most elemental metals but can be violated, for instance, through changes in anisotropy or in the size of the FS, by a field dependence of the anisotropy or of the scattering rate, through changes in the effective mass and by changes in the mobility ratio between different pockets. Furthermore, no QOs have been observed in the cleanest crystals up to the highest field strength (30 T) and down to lowest temperatures (0.35 K). Using the known FS of NbSe_2_ (*35*) and a single degree of freedom (the zero-field scattering rate determined by impurity scattering), we are able to explain the absence of QOs and model the presence of the LMR within the ICM framework. The slope of the *H*-linear MR agrees quantitatively with the model’s prediction, while Kohler scaling and the temperature and disorder independence of the LMR slope are also reproduced. For completeness, limitations of the model and effects beyond the relaxation time approximation are also discussed.

## RESULTS

### Disorder dependence of the high-field MR of NbSe_2_

Figure 1B shows the low-*T* ρ(*T*) curves for five single crystals of NbSe_2_ with a fourfold variation in their residual resistivity ρ_0_ = ρ(*T*_c_). As reported in previous disorder studies, both *T*_c_ and *T*_CDW_ are found to drop with increasing ρ_0_ (*36*–*39*). The CDW opens gaps primarily where parts of the FS are connected by the CDW wave vector $\vec{Q}$ within an individual pocket and most strongly on the inner *K*- and *K*′-barrels (*40*–*42*). The ICM hypothesis applied to NbSe_2_ is that where such gaps open, additional sharp drops in the transport lifetime τ are experienced by the quasiparticles (Fig. 1C). This drop in lifetime is assumed to scale with the strength of the CDW order, disappearing at the CDW transition. An exemplar LMR within the CDW phase is shown in Fig. 1D. Below, we use the FS previously determined via angle-resolved photoemission spectroscopy (ARPES) (*35*) and this scattering lifetime to model the MR of NbSe_2_ using a Boltzmann transport calculation. In addition, to investigate specific limitations of the model, we present calculations that include the proposed FS reconstruction as well as calculations that go beyond the relaxation time approximation.

**Fig. 2.**
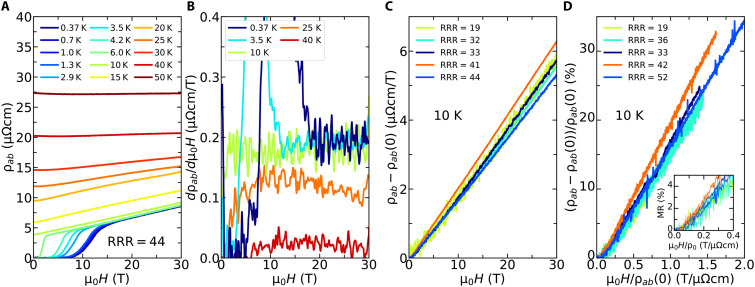
Overview of the magnetoresistance behavior in 2*H*-NbSe_2_. (**A**) Raw MR data for the sample with the largest RRR measured between 0.37 and 50 K. The current is applied along the *a* axis, and the magnetic field is oriented in the *c* direction. We observe a clear *H*-linear MR over an extended field range below 15 K. Above 15 K, the magnitude decreases until the MR effectively vanishes at 50 K. (**B**) Field derivative of the resistivity *d*ρ*_ab_*/*d*μ_0_*H* reveals a robust nonsaturating, *T*-independent LMR below *T* = 15 K that persists up to the highest magnetic fields. Above 15 K, the MR develops a tendency toward saturation. (**C**) MR curves obtained just above *T*_c_ (at 10 K) in the crystals whose zero-field resistivity is shown in Fig. 1B. The high field MR is clearly linear, while the low field MR shows a more quadratic character. The field range of the quadratic behavior depends on the RRR. (**D**) Kohler plot for field sweeps made up to 8 T of crystals cut from the same mother crystals as the high-field samples but with slightly different RRRs. Kohler scaling is obeyed at 10 K as a function of disorder. Inset: zoom of the low-field quadratic part of the MR, showing the robustness of the scaling.

Figure 2A shows a representative set of ρ*_ab_* measurements as a function of applied magnetic field for the sample with RRR = 44, where RRR = ρ*_ab_*(RT)/ρ*_ab_*(*T*_c_). At the lowest temperatures and beyond a magnetic field strength large enough to suppress superconductivity, we observe *H*-linearity up to 30 T. Between *T*_c_ and 15 K, we observe a *H*^2^- to *H*-linear crossover in the MR with a small crossover scale H^*^ between 0.5 and 1.0 T. Above 15 K, the *H*-linearity gradually breaks down, and then above *T*_CDW_, the MR almost vanishes and exhibits more conventional quadratic-to-saturating behavior. Correspondingly, the Hall effect (see fig. S1) is temperature independent and electron-like below 15 K, changes sign at 25 K, and recovers the expected hole-like linear Hall response above ~40 K. At low-*T*, the *H*-linear slope is independent of temperature and has a value of ≈0.18 μΩcm/T (see Fig. 2B). This compares with previous literature values of 0.10 (*43*) and 0.13 μΩcm/T (*27*).

Figure 2C shows Δρ*_ab_* [= ρ*_ab_*(μ_0_*H*) − ρ*_ab_*(0)] of the five measured samples at *T* = 10 K. (The full dataset for all samples can be found in fig. S1). The *H*-linear slope of the MR is found to vary by ±3% despite a fourfold variation in ρ_0_. To properly test Kohler scaling, the *H*^2^-part of the MR should also collapse, which is obscured in Fig. 2C due to the low field scale of the turnover. We therefore performed a low-field study on crystals cut from the same mother crystals as the high field samples but with slightly different RRRs, to test specifically whether the quadratic part of the MR curves follows any type of scaling. The full dataset of these measurements is presented in figs. S2 and S3. We show the Kohler plot of these samples measured at *T* = 10 K in Fig. 2D. In the inset of Fig. 2D, we also show a blow-up of the same Kohler plot, focusing on the low-field response. As can be seen, the curves collapse to within experimental error, implying that the MR in NbSe_2_ does obey Kohler scaling. In the following section, we proceed to model the data to better understand the origin of the LMR in NbSe_2_.

### Modeling the data using ICM

ICM provides a phenomenological description of MR that is insensitive to the microscopic details. This allows us to thoroughly test the model against the experimental data with few degrees of freedom. We follow (*21*) and hypothesize that the predominant effect of the CDW order on the electron dynamics is to turn points on the FS connected by a CDW $\vec{Q}$-vector incoherent or, equivalently, to enlarge their imaginary self-energies into a set of hotspots. The suppression of the carrier lifetime at the hotspots then scales with the strength of the CDW order and impedes cyclotron motion, suppressing QOs and generating LMR.

Even at μ_0_*H* = 30 T and *T* = 0.35 K, QOs associated with the major pockets (with predicted frequencies of 5 to 10 kT) are absent. Under these conditions, they would be expected to trace out a real-space orbit of typical cyclotron radius *R*_c_ ≈ 75 nm based on the known FS of NbSe_2_. This is in stark contrast with the pancake pocket, which has been observed at 2.5 T when *R*_c_ = 225 nm despite being subject to additional scattering in the vortex liquid state (*31*, *32*). We similarly observe QOs from the pancake pocket when we rotate the magnetic field (see fig. S5). The cyclotron radius of the smallest pockets of NbSe_2_ after backfolding and hybridization is expected to be as small as *R*_c_ ≈ 8 nm in our experimental conditions (see the Supplementary Materials for details), yet no QOs are observed in these either. This notable lack of QOs supports the notion that the CDW order in 2*H*-NbSe_2_ forms impedances at specific positions in *k*-space, preventing electrons from completing cyclotron orbits anywhere on the FS (except around the pancake pocket that of itself does not participate in the CDW order).

To test this hypothesis more rigorously, we develop a phenomenological magneto-transport model using the minimal tight-binding expansion of the FS of NbSe_2_ (*35*). Backfolding and hybridization undoubtedly occur, but by themselves do not explain the observations and so we neglect these steps initially to focus on the essential ingredients required to explain both the MR and the absence of QOs. Impedances are placed at locations at which the CDW $\vec{Q}$-vector connects the FS, only keeping intrapocket connections in accordance with (*40*) and noting that the spectral weight observed at other locations on the FS in ARPES remains unchanged (*35*, *44*). The impedances are modeled as locally suppressed lifetimes as shown in Fig. 1C. We use the lifetime for its computational simplicity but emphasize that the results are valid also if the impedances result, e.g., from a removal of spectral weight away from the Fermi energy or from high local effective masses; in all cases, the contribution of the impedances themselves to the total conductivity is negligible (due to shorting effects) and affects the magnetotransport solely by ICM. We use the full Shockley-Chambers tube integral formalism (SCTIF) of the Boltzmann transport equation assuming the relaxation time approximation and neglecting *c*-axis dispersion (*45*, *46*). The only degree of freedom in the model is the isotropic cold scattering time τ across the FS, which we fit to the zero-field resistivity ρ*_ab_*(0).

We start with the most general solution to the Boltzmann transport equation within the relaxation time approximation and without thermal broadening. The resulting formula within the SCTIF is (*45*, *46*)

$$\sigma_{\mathrm{ij}}=\frac{e^{2}}{\text{πℏ}}\int_{\text{FS}}\frac{d^{2}\vec{k}}{{\left(2\pi \right)}^{2}}\frac{v_{i}}{v}\;\int_{0}^{\infty }dtv_{j}\left(-t\right)\text{exp}\left[-\int_{0}^{t}\frac{dt^{\prime }}{\tau \left(-t^{\prime }\right)}\right]$$ (1)

**Fig. 3.**
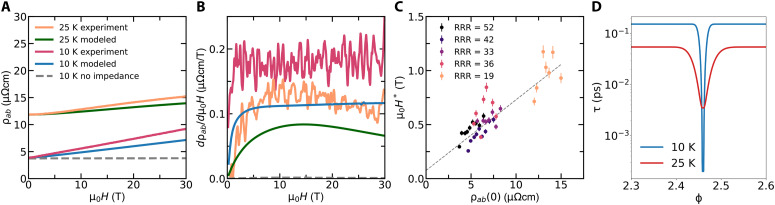
Comparison of the ICM model to the measured data. (**A**) Comparison of the modeled MR response (blue) to the measured data (purple) on the RRR = 44 sample at *T* = 10 K, i.e., well within the *H*-linear regime. The modeled response without impedances (gray) indicates that the MR originates from the impedances and not from the FS itself. A second comparison (green and orange, respectively) is shown at *T* = 25 K (close to *T*_CDW_) where we observe a breakdown of *H*-linearity. This breakdown at high magnetic field can be reproduced by artificially weakening the hotspots such that quasiparticles have a finite probability for breakdown (see the Supplementary Materials for details. (**B**) The derivatives of the curves shown in (A) highlight the linearity and turnover scale. Overall, the model shows good qualitative and reasonable quantitative agreement with the data. (**C**) H^*^ versus ρ*_ab_*(0) obtained by varying both disorder and temperature between 8 and 18 K. Here, H^*^ is extracted as the field value where the derivative *d*ρ*_ab_*/*d*μ_0_*H* reaches 90% of the high-field linear slope. The gray dashed line is a guide to the eye. (**D**) Representative impedances at 10 and 25 K. ϕ refers to the azimuthal angle on the inner Γ pocket. By weakening impedances with increasing temperature toward *T*_CDW_, the observed MR saturation is captured.

Here, σ is the conductivity, indices *i*,*j* run over *x*,*y* (the *k*_z_ corrugation of the FS is neglected), *e* is the electron charge, ℏ is the reduced Planck’s constant, FS is the FS including a sum over the different pockets, τ is the velocity relaxation time, and $\vec{v}∶=\frac{1}{\hbar }{\vec{\nabla }}_{k}\varepsilon_{k}$ is the Fermi velocity defined through the energy dispersion ε*_k_*. The $\vec{k}$ dependence of $\vec{v}$ and τ is implicit for clarity. Time dependence manifests through cyclotron motion, i.e., through $\vec{k}$(*t*). The exponent in Eq. 1 is the probability for a quasiparticle to survive to time *t*. The integrals can be evaluated for arbitrary magnetic field strengths. Temperature dependence arises solely from the *T* dependence of τ. Resistivity is obtained through a full matrix inversion of σ. The model is defined entirely by ε*_k_* and τ($\vec{k}$). We take ε*_k_* from a previous tight-binding fit to ARPES data which contains exactly one hole per unit cell while neglecting the selenium pancake pocket (*35*). τ(*k*) is shown in Fig. 1C for the entire FS and in Fig. 3D for an individual hotspot.

In Fig. 3 (A and B), we directly compare the simulations obtained from our simple model to the experimental data obtained on the sample with RRR = 44. For clarity, we only include curves at *T* = 10 and 25 K. As shown in Fig. 3B at *T* = 10 K, the model generates a robust LMR with a slope of 0.11 μΩcm/T that persists up to the highest fields used in our study. Consistent with the data shown in Fig. 2C, this *H*-linear slope is independent of τ (ρ*_ab_*(0)). Despite the lack of tunability, the model captures well the crossover field H^*^ and reproduces the magnitude of the MR to within 30%.

The LMR slope in ICM is nominally inversely proportional to the cold carrier density (akin to the traditional Hall effect) and robust against changes in scattering time, temperature, disorder, or effective mass as observed experimentally. Upon increasing temperature, the CDW gap decreases, and as a result, the impedances are expected to disappear. Consistent with ICM, the LMR is suppressed near *T*_CDW_. The experimentally observed MR essentially vanishes at 50 K, and the observed and modeled Hall effect matches with expectations from the carrier density (see the Supplementary Materials for details). In the intermediate temperature range just below *T*_CDW_, we anticipate the CDW order to be sufficiently weak that carriers are affected by the CDW, but their cyclotron motion may not be effectively impeded. (Note that given the estimated effective mass in NbSe_2_, this temperature is too high for QOs to be observed.) To incorporate this effect, we introduce a second degree of freedom by artificially weakening the impedances such that magnetic breakdown is possible within the experimentally observable regime. This means that the hotspots are not infinitely strong and there remains a finite probability exp(-*B*_br_*/B*) for charge to maintain cyclotron motion across the impedance. At low *T*, the breakdown field *B*_br_ is much greater than 50 T and decreases toward *T*_CDW_. The result for *T* = 25 K is shown in Fig. 3 (A and B) and reproduces the observations: The MR diminishes in size and saturates in tandem. In other words, the robust LMR has vanished. Note that the tendency of the MR in NbSe_2_ to saturate already at intermediate temperatures is unusual yet is well reproduced by the ICM model. Recent results at low *T* as a function of hydrostatic pressure show that the MR similarly diminishes together with the CDW order (*33*). Collectively, these experimental observations appear to tie the LMR to the CDW order, which is naturally explained by impedances which scale with the CDW order parameter as adopted here. Last, the model naturally reproduces Kohler scaling at low *T* where the isotropic cold scattering rate is the only temperature-dependent parameter.

The turnover scale H^*^ where LMR sets in represents the magnetic field at which cold quasiparticles can traverse between hotspots via cyclotron motion. For this process, we expect the Kohler relation H^*^ ∝ ρ(0) to hold both as a function of temperature (below ~⅔ *T*_CDW_ when impedances appear to be well established and breakdown seems absent) as well as disorder. This relationship, shown in Fig. 3C, agrees well with the model predictions. In the Supplementary Materials, we present a version of the model that also fits the result in absolute units for all temperatures, magnetic fields, and disorder values by incorporating not only the cold scattering rate but also the width and strength of hotspots to account for magnetic breakdown.

Despite its successes, this simple model has some shortfalls that we summarize here while referring the reader to the Supplementary Materials for more details. First and foremost, although ICM describes a simultaneous *H*-linearity in the MR and Hall effect, the sign change in the Hall effect which sets in simultaneously with the LMR remains unexplained. Second, the model relies on the relaxation time approximation and thus neglects contributions to the conductivity from nonzero averaged velocities near the hotspots (*47*). To address these points, we consider in the Supplementary Materials various extensions to the model that include the full FS reconstruction, additional anisotropy in the reconstructed phase from interpocket and interband interactions, and a practical Boltzmann transport framework beyond the relaxation time approximation that was previously applied to quasi–one-dimensional conductors in (*48*). First, we find that the Peierls reconstruction without impedances is insufficient to explain the absence of QOs or the presence of LMR in NbSe_2_. Second, we address any concerns about charge conservation within ICM by developing a fully charge conserving model. Last, we find evidence beyond the relaxation time approximation which favors impedances due to reduced quasiparticle coherence (e.g., through high imaginary self-energy or spectral weight shifted away from the FS) over scattering origins (e.g., domain wall scattering). We stress, however, that further work is necessary to establish the precise microscopic origin of ICM in NbSe_2_.

## DISCUSSION

Comprehensive measurements of the temperature, magnetic field, and disorder dependence of the MR in NbSe_2_ confirm the robustness of the LMR and the concomitant lack of QOs up to higher field strengths (30 T) and down to lower temperatures (0.35 K) than previously reported. Both phenomena have remained unexplained for 50 years. Our minimal ICM model, adapted to the known FS of NbSe_2_, is found to offer a coherent explanation for the robust LMR up to the highest field strength, its magnitude, its onset field H^*^, the vanishing of the MR above *T*_CDW_, the saturation of the MR in the intermediate temperature range, its disappearance under hydrostatic pressure, the disorder independence of the *H*-linear slope, Kohler scaling, the simultaneous linearity of the Hall effect, and the absence of QOs on all pockets except the selenium pancake pocket. The only failure of the model in its current form is its inability to account for the sign change in the Hall effect, which may result from the lack of FS reconstruction in the model.

As highlighted in Introduction, multiple scenarios have been proposed to explain LMR, and the origin of LMR in a particular metal is likely to depend on the specifics of their electronic state. Before discussing the implications of our modeling for the physics of NbSe_2_, therefore, we first describe here what aspects of the data are inconsistent with other models for the *H*-linear MR. While the nonlinear ρ*_yx_*(*H*) in NbSe_2_ hints at multiple carriers, no variant of the multiband Drude model can generate such a robust *H*-linear MR. Models based on random resistor networks (*10*, *49*) or fluctuations in the carrier density (*2*, *19*) can also be ruled out since (i) Δρ is *H*-linear while ρ*_yx_* is nonlinear, and (ii) at low *T*, the magnitude of the MR is essentially constant while ρ*_yx_* changes sign. At the same time, a different disorder dependence would be expected. Previous scanning tunneling microscopy work revealed that CDW domains in NbSe_2_ shrink with increased disorder down to ~5 nm in strongly disordered crystals (*50*). This is expected to enhance the mobility variance and thereby markedly increase the LMR (*49*, *51*), in contrast with the experimental findings (see Fig. 2). Additional assumptions or degrees of freedom would be required for the random resistor network picture to capture all the experimental observables captured by the ICM model. The low field scale for the crossover *H*-linear MR (~1 T) implies that the quantum model of Abrikosov (*12*) is not relevant here. Moreover, the hotspot model of Koshelev (*20*) predicts ρ*_yx_* ~ *H*^2^, while the notion that sharp FS corners account for the *H*-linear MR, as proposed for other CDW compounds (*15*), appears invalid since the robustness of the *H*-linearity up to 30 T implies extremely sharp FS corners and thus reconstruction into smaller pockets, for which one should observe (*13*) QOs and MR saturation well below 30 T in clean NbSe_2_—inconsistent with our results. Last, the robust *H*/*T* MR scaling behavior seen in several strange (*9*) and quantum critical (*4*, *6*) metals indicates that its origin in those systems is tied to the *T*-linear resistivity. In NbSe_2_, by contrast, LMR is observed in a regime where the resistivity does not exhibit *T* linearity.

In the original Peierls picture of CDW order (*52*), nesting of the FS by a wave vector $\vec{Q}$ yields a divergence of the electronic susceptibility. In the presence of even infinitesimal electron-phonon coupling, this is accompanied by a sharp dip (Kohn anomaly) in the phonon dispersion causing a combined electronic and lattice instability and reconstruction of the FS (*53*). In this idealized scenario, there is no additional scattering in the hybridized FS after backfolding, beyond the usual isotropic impurity scattering. In practice, perfect nesting does not exist (*54*). In NbSe_2_, in particular, the applicability of the ideal Peierls picture has long been questioned. It cannot predict the observed CDW vector (*55*), the broad Kohn anomaly (*56*), nor the presence of fluctuations above *T*_CDW_. Models of the CDW order instead require a momentum dependence of the electron-phonon coupling and inelastic processes away from the Fermi level (*41*, *56*, *57*). Our study suggests that impedances to cyclotron motion are an essential extra ingredient to explain both the LMR and the lack of QOs. Last, a scenario based on hotspots formed through strong scattering from (CDW) quantum fluctuations, i.e., due to proximity to a quantum critical point (*4*), seems unlikely here. Thermal variations of the CDW order parameter should be present above *T*_CDW_, be strongest near *T*_CDW_, and be suppressed at lower *T* (*33*, *56*). The LMR in NbSe_2_, meanwhile, is absent above *T*_CDW_, shows breakdown just below *T*_CDW_, and is strongest at low *T*.

An alternative type of fluctuation may lie in the strong coupling nature of the CDW formation (*58*, *59*). Going beyond the idealized Peierls picture, quantitative modeling of the CDW requires renormalizing the bare electrons and phonons with fluctuations beyond the mean field level (*40*, *41*). This results in a strongly momentum-dependent CDW gap but may equally affect the off-diagonal elements of the gap matrix, which determine the lifetime of renormalized electrons. Under the natural assumption that the momentum dependence of the diagonal and off-diagonal elements track one another, the scattering rate is largest at hotspots along the FS connected by CDW wave vectors.

Another possibility is that impedances are formed by scattering from CDW domain walls. Traversing a sharp domain wall can be thought of as quenching from one CDW structure to one shifted by a single lattice spacing. Bloch’s theorem implies that spatially shifting the electronic wave functions adds a momentum-dependent phase factor. For electronic states at hotspots along the FS, the phase shift is guaranteed to yield different electronic states at the FS. The joint density of states available for scattering from CDW domain walls is thus maximized at hotspots, causing their scattering rate to be maximized. Note that although domain sizes larger than the expected cyclotron size at 30 T have been observed in clean NbSe_2_ (see also the Supplementary Materials) (*50*), these do not account for phase slip lines in individual CDW components attached to point defects. The density of such phase slips is determined by the difference between the locally commensurate CDW vector and the incommensurate position of the peak in electronic susceptibility (*40*, *60*, *61*) and may thus be expected to be relatively large and doping independent.

Estimating the CDW correlation length to be ξ ≤ 50 nm and using ARPES results for the CDW gap anisotropy (*35*), the inequality Δ_CDW_ < ℏ*v*_F_/ξ is found to hold for all major FS pockets except for the inner *K* barrel on which the CDW order is strongest. In this regime, it has been suggested that FS reconstruction may be inhibited since the CDW appears glassy to the electrons (*22*). In underdoped cuprates, this inequality is also satisfied, and yet QOs of reconstructed Fermi pockets have been observed (*62*, *63*). More work is required to thoroughly test this mechanism. Last, we point out that two key ARPES studies on NbSe_2_ (*35*, *44*) show a reduction or even vanishing of the quasiparticle spectral weight at the CDW hotspots. At the same time, the backfolded spectral weight is minimal. Certainly, the origin of the impedances found here appear related to the ARPES results. Neither ARPES nor LMR, however, can by themselves reveal the microscopic origin of the spectral weight reduction nor the impedances. Resolving this will require further investigation.

Overall, our study provides indirect but robust evidence for the formation of impedances alongside the usual FS reconstruction in the CDW ordered phase of NbSe_2_. The prototypical nature of NbSe_2_ among (strongly coupled) density wave systems, both charge and spin, indicates that this result may have broader implications. Considering the pnictides (where hotspots from critical spin fluctuations may act as impedances) and optimally doped cuprates (where the edges of Fermi arcs offer a natural source of impedance), the turnover scale of the MR appears entirely insensitive to disorder and scales with *H*/*T*, indicative of some form of strange metallic transport possibly beyond the relaxation time approximation. Nevertheless, these examples illustrate that impedances naturally emerge across strongly correlated electron systems through the *k*-selectivity of a wide variety of correlation effects with distinctive consequences in the presence of a magnetic field (*21*). Understanding how ICM affects the relatively simple Fermi-liquid NbSe_2_ is essential if we are to understand related phenomena in more complex unconventional superconductors.

We conclude by arguing that ICM enables a deeper and simpler understanding of the electrical transport and lack of QOs in NbSe_2_ than is possible via the conventional FS reconstruction scenario. While further work is required to identify the microscopic origin of ICM in NbSe_2_, the ICM scenario presented here offers a promising starting point toward a new paradigm for describing density wave order and correlated electron dynamics.

## MATERIALS AND METHODS

Bulk 2*H*-NbSe_2_ crystals of varying quality, indicated by a varying *T*_c_ and RRR, were obtained from the crystal provider HQ Graphene and cut into bar shapes along the *a* or *b* axis using a razor blade. X-ray diffraction (XRD) measurements indicated that there were no impurity phases present to within experimental resolution present. We tentatively ascribe the disorder to crystal imperfections (e.g., dislocations), point-like impurities, and vacancies created during the growth process. XRD was also used to determine the crystallographic orientation of each crystal. [The crystal structure itself, shown in Fig. 1, was generated using VESTA (*64*)]. Electrical contacts were made in a six-probe Hall bar configuration by fixing 25-μm-diameter gold wires to the sides of the samples using 4929N DuPont silver paste, carefully shorting out the *c* axis to avoid contributions from the out-of-plane resistivity ρ_c_. Note that the data for the RRR = 19 sample in Fig. 2D) are noisier due to a contact that slightly degraded during the experiment. High-field transport properties were measured in a laboratory-built ^3^He system inserted into a 30 T Bitter magnet at the HFML-FELIX using a Keithley 6221 current source and a Stanford Research SR865 lock-in amplifier. The derivatives shown in Fig. 2 were smoothed with a Savitsky-Golay filter of polynomial order 1. Low-field measurements were performed in a Cryogenics cryogen-free measurement system. Modeling was performed using a Python code (see the Supplementary Materials for details).

## Supplementary Materials

### This PDF file includes:

Supplementary Text Figs. S1 to S14 Tables S1 and S2 References
