# Supplementary material for: H-linear magnetoresistance in NbSe2 due to impeded cyclotron motion
Source: Sci Adv. 2026 Apr 29;12(18):eaea6029. doi: 10.1126/sciadv.aea6029 (PMC13127583; doi:10.1126/sciadv.aea6029)
Supplement: Supplementary file 1 — Supplementary Text Figs. S1 to S14 Tables S1 and S2 References [file sciadv.aea6029_sm.pdf]

Science Advances
AAAS

Supplementary Materials for

H-linear magnetoresistance in $NbSe_2$ due to impeded cyclotron motion

Arwin Kool *et al.*

Corresponding author: Arwin Kool, arwin.kool@ru.nl; Nigel E. Hussey, n.e.hussey@bristol.ac.uk;
Roemer D. H. Hinlopen, roemer.hinlopen@mpsd.mpg.de

Sci. Adv. **12**, eaea6029 (2026)
DOI: 10.1126/sciadv.aea6029

This PDF file includes:

Supplementary Text
Figs. S1 to S14
Tables S1 and S2
References

Supplementary Text

High field dataset

In the main article, we discussed and showed the full temperature (T) dependence of the magnetoresistance (MR) of the $NbSe_2$ sample with a residual resistivity ratio RRR = 44. For our disorder study, we measured samples cut from four more crystals with varying RRR, whose R-T curves are shown in Fig. 1**B** of the main article. All samples were cut along the a- or b-axis.

Fig. S1 shows the full T-dependence of the MR for all five of these crystals measured between 0.3 K and 50 K. The top five panels show the in-plane resistivity ρ_{ab} as a function of field. All 5 samples show the same qualitative behavior of the MR as the sample discussed in the main article. Furthermore, the absolute value of the resistivity increases with decreasing RRR, consistent with Matthiessen's rule. The extra structure at the superconducting phase transition in these samples is attributed to increased inhomogeneity, leading to a variation of H_{c2} inside each sample. Panels **F**-**J** of Fig S1 show selected first derivatives of the MR curves in order to emphasize the robustness of the H-linear slope over a wide temperature and field range, despite a 4-fold change in the residual resistivity ρ_0.

Panels **K**-**M** of Fig S1 show the Hall resistivity ρ_{yx} measured on four of the five samples studied in high field. (There is no data shown for the sample with RRR = 19, as it did not have a working Hall contact.) We see that at low T, ρ_{yx} exhibits highly non-linear behavior, suggesting that charge carriers from multiple Fermi pockets are participating in the transport. The high-field Hall resistivity is negative and T-independent up to 10 K, after which it gradually diminishes before changing sign at $T \approx 25$ K. At higher temperatures, ρ_{yx} remains positive and tends towards linearity, becoming fully H-linear above 40 K. We note that the sign change coincides with the breakdown of H-linearity in all samples, and the Hall resistivity becoming completely linear coincides with a recovery of a more conventional MR. Curiously, the sign change in the Hall coefficient R_H happens for all samples at roughly 2/3 T_{CDW}, where T_{CDW} is the transition temperature into the charge density wave (CDW) state that changes slightly under the influence of disorder. As R_H changes sign at the same temperature where H-linearity is lost, we interpret the sign change as a consequence of the weakening of CDW order. Because of this, the impedances on the Fermi surface (FS) weaken, and we slowly recover the high-T hole-like FS of $NbSe_2$. Our data suggest that the impedances are caused by the CDW order which strengthens substantially down to 15 K.

Low-field dataset

To assess the dependence of the crossover field $\mu_0 H^*$ on the zero-field resistivity $\rho_{ab}(0)$, and to more closely map the breakdown of H-linearity upon increasing temperature, we performed a set of low field measurements up to 8 T in an automated cryogen-free measurement system (CFMS) from *Cryogenics Ltd*. For this study, we reused the samples with RRR = 33 and RRR = 19, but with new electrical contacts. For the other three batches, from which the samples with RRR = 44, 41 and 32 originate, three new samples were cut from the same mother crystals. The new samples have RRR values of 52, 42 and 36, respectively. The low field data were taken at intervals of 1 K or 2 K. All samples were contacted in a Hall bar geometry to allow for simultaneous measurements of ρ_{ab} and ρ_{yx}. All samples were again cut along the a- or b-axis.

Fig. S2 shows $\rho_{ab}(T)$ of these 5 samples. We again see that Matthiessen's rule is satisfied at low T, and that we have a variation in the residual resistivity ρ_0 by a factor of around 4. The

field-dependent data obtained with the CFMS are shown in Fig. S3. In panels **A**-**E** of Fig. S3, we show $\rho_{ab}(H)$ for all five measured samples. All curves were measured between 6 K and 36 K. To within experimental error, the measured resistivities are consistent with the high-field data reported in Fig. S1. In panels **F**-**J** of Fig. S3, we show the corresponding field derivatives. From these curves, we can see that the crossover scale $\mu_0 H^*$, defined as the field where $d\rho_{ab}/d\mu_0 H$ reaches 90% of its high field value, is always around 1-2 T, and increases with increasing $\rho_{ab}(0)$. Furthermore, we can clearly see the gradual breakdown of the *H*-linearity with increasing temperature, which typically occurs around $T = 18$ K, after which the slope also diminishes. Hence, above this temperature scale, CDW correlations are sufficiently suppressed to allow electrons to tunnel through the impedances.

Panels **K**-**O** of Fig. S3 show the Hall resistivity measured on the 5 samples with varying *RRR* values up to 8 T. Note that these curves have been smoothed using a Gaussian smoothing algorithm. The Hall resistivity ρ_{yx} is temperature independent up to 15 K, after which it slowly starts to decrease in magnitude. Around 25 K, R_H changes sign, indicated by a $\rho_{yx}(H)$ which is zero over almost the entire field range. For $T > 25$ K, ρ_{yx} is positive and becomes progressively more linear. Above T_{CDW}, ρ_{yx} becomes fully *H*-linear and independent of temperature within the studied temperature range. Again, the magnitude of the Hall effect is consistent with the high field data to within experimental error.

We see here that the sign change in R_H and the breakdown of *H*-linearity do not occur at the exact same temperature. Nevertheless, we can still ascribe the sign change in R_H to a weakening of the CDW order. In panels **F**-**J** of Fig. S3, we see that although *H*-linearity is lost above 18 K, there is still a sizable MR between 18 K and 25 K. We know from the results of the modelling that the conventional orbital MR of the high-*T* FS of $NbSe_2$ is small compared to the magnitude of the observed LMR, suggesting the presence of a remnant contribution from ICM in this temperature range. Furthermore, 18 K coincides with the temperature where R_H starts to strongly deviate from its low-*T* value. Therefore, the temperature at which R_H changes sign appears to coincide with the temperature at which tunneling through the impedances on the FS caused by the CDW order is possible. However, the Hall effect appears more sensitive to such breakdown than the MR. ICM shows a similar dependence on FS topology as the Hall effect.

Longitudinal magnetoresistance

The concept of impeded cyclotron motion assumes that the MR is predominantly orbital in nature. If this is the case, the MR is expected to vanish for a field parallel to the current, since in this configuration the charge carriers do not experience a Lorentz force. To test this, we also measured the MR of the high-field samples with **H**//*I*//*ab* at $T = 0.37$ K and 4.2 K (deep within the ICM regime) and at $T = 30$ K, i.e. close to where the CDW vanishes and ICM fully breaks down.

Figure S4 shows the resultant data. A finite MR is still visible in all samples, although its magnitude is much reduced compared to the transverse MR (**H**//*c*). This nonzero MR most likely arises from a misorientation of the samples in the magnetic field. The angle with respect to the applied field can be minimized by monitoring the signal of a Hall bar as the probe is rotated, though a small, residual angle (~3°) between the sample orientation and the Hall bar is sufficient to explain the observed MR. We therefore conclude that the low-*T* MR response in $NbSe_2$ is indeed orbital in nature.

Quantum oscillations

A striking feature in the MR of $NbSe_2$ is the absence of quantum oscillations (QOs) from any of its major pockets even in the cleanest samples (*31*,*32*). These are expected at frequencies of 5-10 kT without and 0.05-2 kT with FS reconstruction (see Table S1 below). Indeed, no QOs are visible in any of the data shown in Fig. 2, Fig. S1 and Fig S3. The only pocket known to exhibit QOs (through the de Haas-van Alphen or dHvA effect), is the pancake-like pocket centered around the Γ-point in the Brillouin zone (BZ). Here we show that we also observe, for the first time, Shubnikov-de Haas oscillations arising from this pocket, i.e. in the electrical resistivity, in a sample with *RRR* = 39. Note that on this sample, the *c*-axis was not properly shorted out, so the *c*-axis possibly also contributes to the transport. For this reason, we report here only resistance data, not resistivity data.

Fig. S5**A** shows MR curves $R(B)$ between 0.9 K and 18 K. We again see a robust *H*-linear MR that spans a wide temperature and field range. Furthermore, we see no QOs down to the lowest temperatures. In Fig. S5**B** we show the angle dependence of $R(B)$ versus field measured at 1.35 K, where the angle θ is defined as 0° for a magnetic field oriented along the *c*-axis, and θ=90° for the field parallel to the current. When we rotate the field away from the *c*-axis, we observe an increase in the MR up to roughly 40°, after which the MR decreases in magnitude. Furthermore, for angles larger than 50°, we observe the onset of QOs which increase in magnitude with increasing angle. We plot the derivative $dR/d(1/B)$ versus $1/B$ in Fig. S5**C** to remove the background from the QOs. In the inset, we plot the angle dependence of the QO frequency, which shows a clear decrease with increasing angle θ. These angular dependences are representative of a strongly ellipsoidal orbit in the *ab*-plane whose semi-minor axis grows rapidly when the field is rotated out of the plane. This confirms that we are observing QOs from the Se pancake-like pocket at the Γ-point of the first BZ, consistent with the observations in Ref. (*32*). This implies that the absence of QOs from the pockets participating in the CDW order is not a matter of sample quality, but rather has a more intrinsic physical mechanism behind it.

Conductivity models

Below we report on a variety of theoretical models with direct relevance to the origin of LMR in $NbSe_2$. In section A, we discuss the conductivity formula and form for the hotspots used for the ICM model in the main text. We detail the insensitivity of the model to the width and strength of hotspots at low T and our approach to incorporate magnetic breakdown just below T_{CDW}.

In section B, we explore the expectations within the Peierls picture. First, we establish the reconstructed FS itself as well as the myriad of expected QOs. Secondly, we evaluate the conductivity to arbitrary magnetic field strength on this complex FS in both the isotropic lifetime and isotropic-ℓ regimes. We find neither version can explain the extended *H*-linear regime with robust slope nor absence of quantum oscillations in experiment.

Finally, in section C we explore effects beyond the relaxation time approximation (RTA). This requires introducing a new framework expanding on previous work by the present authors in quasi-1D (*48*). We use this framework to show 1) small-angle scattering reduces the *H*-linear slope, 2) present an impeded cyclotron motion model with strict charge conservation and 3) take into account the non-colinear velocity at hotspots. The latter results suggest that termination of quasiparticle coherence at hotspots (e.g. through large imaginary self-energy or removal of spectral weight away from the Fermi level) explains the data better than scattering hotspots (e.g.

by domain walls). We emphasize that none of the extra models presented here substantially improve upon the simple, phenomenological model presented in the main text. They serve to confirm the necessity of impeded cyclotron motion and the robustness of the results to a wide range of corrections.

A. Relaxation time model of impeded cyclotron motion

We now detail the model used to create the theory results shown in the main article. In order to model the impedances to cyclotron motion, we use the most general solution to the Boltzmann transport equation within the RTA and neglect thermal broadening. The resulting general formula was also used to propose impedances to cyclotron motion in Ref. (*21*) and is known as the Shockley-Chambers Tube Integral Formalism (SCTIF) (*45*,*46*):

$$\sigma_{ij} = \frac{e^2}{\pi\hbar}\int_{\mathrm{FS}}\frac{\mathrm{d}^2\vec{k}}{(2\pi)^2}\frac{v_i}{v}\int_0^\infty \mathrm{d}t v_j(-t)\exp\left(-\int_0^t\frac{\mathrm{d}t'}{\tau(-t')}\right) \tag{S1}$$

Here, σ is the conductivity, indices i,j are x,y (since the k_z corrugation of the FS is neglected), e is the electron charge and positive, $\hbar$ the reduced Planck's constant, FS refers to the FS including a sum over the different pockets, $\vec{v}$ is the Fermi velocity with implicit $\vec{k}$-dependence and τ the scattering rate in the RTA again with implicit $\vec{k}$-dependence. Spin degeneracy is included in the formula. The exponent in Eq. (S1) is the probability for a quasiparticle to survive to time t under changing scattering lifetime as the quasiparticle traverses the FS. Time evolves the position of a quasiparticle on the FS due to cyclotron orbital motion $\vec{k}(t)$ and thereby changes both $\vec{v}$ and τ. The integral is evaluated without approximation for the strength of the magnetic field. The velocity is defined through the energy dispersion as usual via

$$\vec{v} := \frac{1}{\hbar}\vec{\nabla}_k\varepsilon_k \tag{S2}$$

The energy dispersion used in the calculations is the tight-binding expansion of Ref. (*35*) based on detailed ARPES results. This tight-binding parameterization satisfies charge conservation, meaning that the combined bands contain one hole per unit cell. The pancake pocket at the Γ-point is neglected throughout for it only contributes significantly to the z-component of the conductivity.

The final ingredient to fully define Eq. (S1) is the scattering lifetime across the FS. We pragmatically choose the form $\tau(\vec{k}) = \tau_c + \alpha(\vec{k})(\tau_h - \tau_c)$. Here, τ_c is the isotropic cold scattering time and the main degree of freedom of the model. τ_c is fit to the zero-field resistivity in experiment and varies between 0.1 and 0.3 ps, dependent on temperature. LMR is reached when cold charge can traverse between subsequent hot spots. Thus, τ_c is inversely proportional to the turnover scale of the LMR. For τ_h we seek a form which: 1) is local in k-space; 2) has no discontinuity; 3) is nevertheless sufficiently strong to impede cyclotron motion: 4) contains minimal degrees of freedom. We note that once the probability for charge to traverse a hotspot is below ~1% (in order to prevent magnetic breakdown), the absolute magnitude of τ_h is irrelevant. The difference between the addition of scattering times or rates is similarly irrelevant to the outcome of the model. We require a smooth τ_h rather than a delta function such that a strictly H^2 MR remains at zero field as also found in Ref. (*21*). In the end, charge at the hotspots themselves are shorted out (contribute negligibly to the conductivity) and the MR is generated by the cold charge subject to

essentially isotropic scattering τ_c driven into the impedances. We use a Gaussian form for $\alpha(\vec{k})$ to satisfy these requirements with minimal degrees of freedom.

$$\tau(\vec{k}) = \tau_c + \alpha(\vec{k})(\tau_h - \tau_c) \tag{S3}$$

$$\alpha(\vec{k}) = \exp\left(-\frac{min\left(\varepsilon^2_{\vec{k}+\vec{Q}}\right)}{2\varepsilon^2_{\Delta}}\right) \tag{S4}$$

Here, $\varepsilon_{\vec{k}+\vec{Q}}$ is the energy after translation by $\vec{Q}$ where $\varepsilon_{\vec{k}} = 0$ at the Fermi level. The minimum then selects the value closest to the Fermi level from the 12 different options (3 unique $\vec{Q}$ as well as $\pm\vec{Q}$ for each and 2 bands). The central result in the main article further selects among these options in order to only allow for intra-pocket connections within individual bands. This choice is dictated by ARPES results in Refs. (*35,44*), which both find spectral weight reduction only where intra-pocket connections are formed by the CDW order. Later theoretical work (*41*) explained also that interband phonon coupling is expected to be negligible. We have neglected the mass enhancement factor measured in (*35*) for the angle dependence does not cover the full Fermi surface and lacks a temperature dependence to perform consistent calculations. However, we do not expect this to have meaningful impact on the results. The reason is that our model cannot differentiate lifetime or mass individually, only their ratio τ/m^* -- i.e. the mean free path. Thus, the isotropic mass enhancement is naturally absorbed into the cold scattering rate when we fit $\rho_{ab}(H = 0)$. Meanwhile, the effective mass peaks are absorbed into the hot spots. Details about this procedure can be found in Ref. (*65*). The shape of the hotspot and dependence of the result on the value of τ_h is shown in Fig. S6. The resulting shape of the hotspot used to model the data at T = 10 K and 25 K for the RRR = 44 sample is shown in Fig. 3**D** of the main article. As shown in Fig. S6, above a certain hotspot strength the probability for charge to survive the hotspot is irrelevant within the experimentally accessible field range. Above this threshold (below ~1% probability) the strength of the hotspot is irrelevant and cyclotron motion is fully impeded. In this regime, τ_h may well vanish and is therefore no longer a fitting parameter. However, when the hotspots are weakened such that breakdown is possible, the MR saturates and the saturation field scale sensitively depends on τ_h, which is the case for the 25 K model data shown in Fig. 3**B** of the main text.

The parameter ϵ_Δ controls the width of the hotspots. At high values (broad hotspots, $\epsilon_\Delta >$ 10 meV) we find a noticeable increase in the turnover scale of the MR, see Fig. S7. Note that this trend is the opposite of the single-band single-hotspot model presented in Ref. (*21*), where increasing the hotspot width results in a small decrease in H^* due to the reduced distance between subsequent impedances. This difference is a consequence of the multiband character of $NbSe_2$ and the presence of variable inter-hotspot distances.

It is important to stress that the model uses the fewest possible degrees of freedom to model the formation of impedances on the FS of $NbSe_2$ while using the most general solution to the Boltzmann transport equation within the RTA. The ingredients for the model are the FS shape (determined by the tight-binding coefficients from ARPES (*35*)), locations of the impedances (determined by the known $\vec{Q}$), the cold scattering lifetime τ_c (fit to the zero-field resistivity), the width of the impedances (irrelevant as long as magnetic breakdown is absent) and the strength of the impedances (irrelevant as long as magnetic breakdown is absent). Note that among these ingredients, only one (τ_c) constitutes a variable fitting parameter at T < 15 K.

Eq. (S1) is evaluated without further approximations. In particular, $\vec{v}$ and τ update continuously along the full cyclotron orbit $\vec{k}(t)$. This holds true in the zero-field limit when no cyclotron motion occurs all the way to the high-field regime beyond magnetic breakdown when cyclotron motion spans multiple orbits and τ changes by orders of magnitude repeatedly. Computationally, this poses a challenge. A custom-made adaptive Simpson integration routine was developed for the t integral in time-ordered fashion. Using time-ordered integration, the probability for a quasiparticle to survive (the exponential term in Eq. (S1)) can be updated at a computationally low cost and the timestep dynamically updated. Note that although we chose to compute the results in the time basis, formally transforming these integrals to φ using $d\varphi = \omega_c dt$ does not change the result. The code is in quantitative agreement with Drude theory under a magnetic field and reproduces the results in Ref. (*21*) to numerical accuracy (better than 1 in 10^6). The resistivity is obtained a full matrix inversion of the conductivity tensor for all calculations. Further details and code are available in Ref. (*65*).

In the main article, we only show fitting at $T = 10$ K (with fully-formed impedances and 1 fitting parameter, τ_c) and for $T = 25$ K (with breakdown and 3 parameters: τ_c, τ_h and ϵ_Δ). While these minimal parameterizations led to a fit with a 30% deviation from the measured LMR slope, they nevertheless highlight the fact that with increasing T, the impedances will likely weaken and lead to magnetic breakdown.

To gain more detailed insight, we consider here a more precise fit to the data. The full temperature range for the $RRR = 41$ sample (with the highest signal to noise ratio) is shown in Fig. S8. In order to produce a precise fit, we resort to renormalizing our observed resistivity values to fit the H-linear slope of the model, since the model itself has no degrees of freedom. We find that a good fit is obtained by renormalizing to a room temperature resistivity value to 110 $\mu\Omega$cm. This is a significant change of about 40 %, but the spread of experimental resistivity data is large due to geometric uncertainty and the quoted room temperature resistivity is in accordance with Ref. (*37*).

After this renormalization, we fit the data as shown in Fig. S8 with parameters listed in Table S2. As the onset of breakdown suggests, we find that the hotspots weaken with increasing T. Indeed, τ_c and τ_h converge close to T_{CDW}. Meanwhile, we find that the hotspots broaden substantially. This quantitatively explains the low H^2 coefficient (high turnover scale H^*) at low magnetic fields at $T = 25$ and 30 K (deviating from T-dependent Kohler's rule). Thus, ϵ_Δ grows large towards T_{CDW}, an opposite T-dependence to what is expected of an order parameter. Instead, we find this trend resonates with the broad softening of phonon modes near Q_{CDW} (as opposed to a sharp Kohn anomaly) observed experimentally in Ref. (*56*). Our results indicate that below T_{CDW}, Q_{CDW} gradually sharpens or locks in, reducing the size of hotspots (i.e. ϵ_Δ). Such a description suggests that the coherence length of the phonons relevant in the strong coupling limit is the key driver to hotspot size rather than the CDW gap itself.

The nominal disorder dependence of the model follows Kohler's rule by changing τ_c to match changes in the residual resistivity. Indeed, this is what is observed experimentally as shown in Fig. 3C of the main article and Fig. S8E-F). We note that the next-leading-order effect of disorder is expected to be a reduction in phonon lifetimes, manifesting as a wider Q_{CDW} creating broader hotspots. In our model, ϵ_Δ would increase in disordered crystals leading to a higher turnover scale H^* in experiment (see Fig. S7). Given the reduction in T_{CDW} between our clean and most disordered crystal of 10 %, we must conclude that the experimental data in Fig. S8E) is not accurate enough to make any conclusions on weak deviations from Kohler scaling of this sort.

B. Relaxation time models incorporating the reconstruction

The Peierls reconstruction model forms the basis of our understanding of CDW order. In this section, we present in-depth calculations which include this reconstruction. To the best of our knowledge, such an endeavor has not been attempted before. The key finding is that the Peierls reconstruction alone is insufficient to explain the experimental results. In particular, these calculations show that without lifetime effects in addition to the standard Peierls model 1) a robust non-saturating H-linear MR cannot be obtained, and 2) QOs are expected to appear under the experimental conditions. Only the sign change of the Hall effect is captured.

We start by implementing the CDW reconstruction on the ARPES-derived FS of $NbSe_2$ (*35*). To proceed, the pancake pocket as well as the small incommensuration of the CDW order are neglected. Practically, we fold back the FS with $\vec{Q}_1$ = ⅔ΓM as well as $\vec{Q}_2$ and $\vec{Q}_3$ obtained by a 120°and 240° counter-clockwise rotation of $\vec{Q}_1$, respectively. Given that $3\vec{Q} = \Gamma$, we also fold using $2\vec{Q}_1 = -\vec{Q}_1$, $-\vec{Q}_2$ and $-\vec{Q}_3$. In order to obtain a closed group of Q-vectors under addition, we must also include $\vec{Q}_4 = \vec{Q}_1 + \vec{Q}_2$ and $-\vec{Q}_4$. In total this adds up to nine copies of the FS for each of the two bands, resulting in an 18×18 size matrix to construct the Hamiltonian. Copies which are separated by $\pm\vec{Q}_1$, $\pm\vec{Q}_2$ or $\pm\vec{Q}_3$ open an intraband (Δ) or interband (Ω) gap, while higher-order couplings are neglected. The final Hamiltonian is as follows:

$$\hat{H} = \begin{pmatrix}
\varepsilon^1_{\vec{0}} & \Delta & \Delta & \Delta & \Delta & \Delta & \Delta & 0 & 0 & 0 & \Omega & \Omega & \Omega & \Omega & \Omega & \Omega & 0 & 0 \\
\Delta & \varepsilon^1_{\vec{Q}_1} & \Delta & \Delta & 0 & 0 & \Delta & \Delta & \Delta & \Omega & 0 & \Omega & \Omega & 0 & 0 & \Omega & \Omega & \Omega \\
\Delta & \Delta & \varepsilon^1_{-\vec{Q}_1} & 0 & \Delta & \Delta & 0 & \Delta & \Delta & \Omega & \Omega & 0 & 0 & \Omega & \Omega & 0 & \Omega & \Omega \\
\Delta & \Delta & 0 & \varepsilon^1_{\vec{Q}_2} & \Delta & \Delta & 0 & \Delta & \Delta & \Omega & \Omega & 0 & 0 & \Omega & \Omega & 0 & \Omega & \Omega \\
\Delta & 0 & \Delta & \Delta & \varepsilon^1_{-\vec{Q}_2} & 0 & \Delta & \Delta & \Delta & \Omega & 0 & \Omega & \Omega & 0 & 0 & \Omega & \Omega & \Omega \\
\Delta & 0 & \Delta & \Delta & 0 & \varepsilon^1_{\vec{Q}_3} & \Delta & \Delta & \Delta & \Omega & 0 & \Omega & \Omega & 0 & 0 & \Omega & \Omega & \Omega \\
\Delta & \Delta & 0 & 0 & \Delta & \Delta & \varepsilon^1_{-\vec{Q}_3} & \Delta & \Delta & \Omega & \Omega & 0 & 0 & \Omega & \Omega & 0 & \Omega & \Omega \\
0 & \Delta & \Delta & \Delta & \Delta & \Delta & \Delta & \varepsilon^1_{\vec{Q}_4} & 0 & 0 & \Omega & \Omega & \Omega & \Omega & \Omega & \Omega & 0 & 0 \\
0 & \Delta & \Delta & \Delta & \Delta & \Delta & \Delta & 0 & \varepsilon^1_{-\vec{Q}_4} & 0 & \Omega & \Omega & \Omega & \Omega & \Omega & \Omega & 0 & 0 \\
0 & \Omega & \Omega & \Omega & \Omega & \Omega & \Omega & 0 & 0 & \varepsilon^2_{\vec{0}} & \Delta & \Delta & \Delta & \Delta & \Delta & \Delta & 0 & 0 \\
\Omega & 0 & \Omega & \Omega & 0 & 0 & \Omega & \Omega & \Omega & \Delta & \varepsilon^2_{\vec{Q}_1} & \Delta & \Delta & 0 & 0 & \Delta & \Delta & \Delta \\
\Omega & \Omega & 0 & 0 & \Omega & \Omega & 0 & \Omega & \Omega & \Delta & \Delta & \varepsilon^2_{-\vec{Q}_1} & 0 & \Delta & \Delta & 0 & \Delta & \Delta \\
\Omega & \Omega & 0 & 0 & \Omega & \Omega & 0 & \Omega & \Omega & \Delta & \Delta & 0 & \varepsilon^2_{\vec{Q}_2} & \Delta & \Delta & 0 & \Delta & \Delta \\
\Omega & 0 & \Omega & \Omega & 0 & 0 & \Omega & \Omega & \Omega & \Delta & 0 & \Delta & \Delta & \varepsilon^2_{-\vec{Q}_2} & 0 & \Delta & \Delta & \Delta \\
\Omega & 0 & \Omega & \Omega & 0 & 0 & \Omega & \Omega & \Omega & \Delta & 0 & \Delta & \Delta & 0 & \varepsilon^2_{\vec{Q}_3} & \Delta & \Delta & \Delta \\
\Omega & \Omega & 0 & 0 & \Omega & \Omega & 0 & \Omega & \Omega & \Delta & \Delta & 0 & 0 & \Delta & \Delta & \varepsilon^2_{-\vec{Q}_3} & \Delta & \Delta \\
0 & \Omega & \Omega & \Omega & \Omega & \Omega & \Omega & 0 & 0 & 0 & \Delta & \Delta & \Delta & \Delta & \Delta & \Delta & \varepsilon^2_{\vec{Q}_4} & 0 \\
0 & \Omega & \Omega & \Omega & \Omega & \Omega & \Omega & 0 & 0 & 0 & \Delta & \Delta & \Delta & \Delta & \Delta & \Delta & 0 & \varepsilon^2_{-\vec{Q}_4}
\end{pmatrix} \quad \text{(S5)}$$

Here, $\varepsilon^a{}_b$ is shorthand for $\varepsilon^a(\vec{k}-\vec{b})$ for formatting reasons. a = 1,2 refers to the band in the ARPES tight binding model and $\vec{k}$-$\vec{b}$ refers to the k-position where the dispersion is evaluated, effectively shifting the dispersion by $+\vec{b}$. Using Δ = Ω = 3 meV the resulting FS and new BZ are shown in Fig. S10**A** below. An overview of the properties of the various pockets before and after reconstruction are listed in Table S1.

Next, we compute the conductivity of this reconstructed FS. The simplest possible analysis is to neglect all the distorted shapes and apply Drude theory, treating each pocket as a perfect circle. This approximation appears crude in light of the pronounced anisotropy in the shapes shown in Fig. S10**A**. However, we will show with detailed calculations below that this simplistic calculation nevertheless captures a number of properties of the magnetotransport that are tied directly to the semimetallic nature and to which the FS anisotropy is largely irrelevant. Thus, this simplistic first analysis serves to understand which aspects of the model result from semimetallicity and which from FS anisotropy. The conductivity contribution of each pocket is then simply $\sigma_{xx} = \sigma_{yy} = ne^2\tau/m^*/(1 + \omega_c^2\tau^2)$ and $\sigma_{xy} = -\sigma_{yx} = \omega_c\tau\sigma_{xx}$. For each pocket, we use the number of copies in the BZ as well as carrier density n, carrier type (sign of $\omega_c\tau$) and effective mass m^* listed in Table S1. $\omega_c = qB/m^*$ is the cyclotron frequency where $q = \pm e$ is signed. The only degree of freedom is an isotropic $\tau = 0.36$ ps for all pockets, consistent with a realistic zero-field resistivity of 3.0 $\mu\Omega$cm at 10K. This lifetime is comparable to the value used without reconstruction due to the low fraction of the density of states gapped by the CDW order. After matrix inversion, the resulting magnetotransport is shown in Fig. S9.

We point out a number of aspects associated with this result. Firstly, the Hall effect is negative as a result of the 3:1 electron:hole ratio. Secondly, the size of the negative Hall effect deviates from experiment as the theoretical result vanishes at low magnetic field and is double the experimental value at high magnetic fields indicating fewer holes are actually present at the Fermi level. Thirdly, the MR is of the correct order of magnitude but saturates and the quadratic regime extends to a field strength that is too high. The tendency of the semimetallic MR to grow large despite only a small admixture of holes is well known (*13*). Finally, there is no extended, non-saturating *H*-linear regime in the MR curve.

Next, we perform detailed resistivity calculations on the reconstructed FS. We employ the full SCTIF formalism of Eq. (S1) as above for impeded cyclotron motion. However, some of the reconstructed pockets are sufficiently anomalously shaped that they backtrack in φ during their orbits relative to any origin placed inside. A single φ value may thus refer to multiple points on the orbit and at inflection points any φ-derivative diverges as a result of this loss of bijectivity. We therefore embrace the fully k-space path-length approach without any underlying parameterization using azimuthal angle φ for all integrals. We also rely solely on the mean free path and FS shape. No reference to velocity, ω_c, τ or $\omega_c\tau$ is required. We achieve both aims by expanding the Ong formalism (*66*) beyond the low-field limit. Without approximation, we substitute time in favor of path- length in k-space along the orbit k_o using the equations of motion in Eq. (S1). We use the fact that velocity and magnetic field are always orthogonal in the present context to simplify this step $\hbar\dot{k_o} = qvB$, where subscript o refers to the path-length along the orbit towards the past:

$$\sigma_{ij} = \frac{e^2}{\pi\hbar}\int_{\mathrm{FS}} \frac{\mathrm{d}^2\vec{k}}{(2\pi)^2}\frac{\ell_i}{\ell}\int_0^\infty \mathrm{d}k_o\hbar\frac{\ell_j(k_o)}{eB\ell(k_0)}\,\exp\left(-\int_0^{k_o}\frac{\hbar dk_o'}{eB\ell(k_0')}\right) \tag{S6}$$

Here, ℓ is the size of the mean-free-path and ℓ_i the x or y component. This formula allows us to compute the conductivity using solely the FS shape and mean-free-path vector across it (whose direction is set perpendicular to the local curvature). By using solely the information that is absolutely required, we are able to keep the number of degrees of freedom to an absolute minimum. On top of this, we again employ time-ordered custom Simpson integration but now in terms of k_o to enable efficient integration without assumption about the magnetic field strength.

Although $B = 0$ is strictly ill-defined, the limit $B \rightarrow 0$ is exactly the same as the SCTIF and in practice we are able to maintain numerical accuracy down to 10^{-5} T.

To fully define the model, we employ an isotropic ℓ across the reconstructed FS. Because the magnetotransport is ultimately determined by the shape of the FS and the mean-free-path vector across it, comparing an isotropic ℓ to the Drude model essentially highlights the impact of the complex Fermi pocket shape formed by the reconstruction. An isotropic ℓ is the nominal expectation for the scattering rate at low T where only elastic scattering off point-like impurities is relevant. The result is shown in Fig. S10.

Comparing the results with the Drude model (Fig. S9), we find that the high-field regime above about 15 T is similar. This is expected as high $\omega_c\tau$ means that quasiparticles traverse most of the Fermi pocket before decaying, washing out the influence of anisotropy. While the high-field regime is similar to the Drude model, the anisotropy of the FS leaves a pronounced mark on the low-field MR. We find a steep quadratic MR up to ~0.4 T compared to the much more gradual quadratic MR up to ~7 T in the Drude scenario. This strong modification of the low-field MR thus emerges as the leading candidate to explain the low turnover scale H^* found experimentally compared to the simple theoretical impeded cyclotron orbital model in the main article.

To further differentiate the impact of the FS anisotropy versus the electron-hole mixture, we change all pockets to electron-like and show the resulting magnetotransport in the dashed lines of Fig. S10. No other changes were made. The anisotropy of the FS shape indeed determines the entire MR below 3 T, yet without semimetallicity the MR would quickly saturate above 5 T. Thus, the sharp corners in the FS cannot explain the non-saturating *H*-linear MR up to 30 T observed in experiment. Indeed, Pippard's classic work on magnetotransport clearly outlines that the linear MR from sharp FS corners saturates when quasiparticles can traverse between corners, i.e. when $\omega_c\tau$ approaches the corner-to-corner distance. This saturation field for FS corners is therefore considerably lower than the magnetic field where QOs appear, which requires complete cyclotron orbits. The origin of the extended region of quasi-linearity in Fig. S10 compared to the Drude model (Fig. S9) thus arises from two separate peaks: A 1-5 T MR peak due to FS anisotropy, and a 7-20 T peak due to the semimetallic nature of the FS. There is no *a priori* reason why these two quasi-linear slopes need to be the same and some deviation from *H*-linearity in the transition region would thus be expected in $d\rho_{ab}/d\mu_0 H$. However, experimentally the *H*-linear slope appears entirely constant as a function of magnetic field, even well above 20 T using derivatives. We point out that the vanishing Hall coefficient at low magnetic fields and its large value at high magnetic fields both suggest the theoretical model overestimates the number of holes substantially, which would reduce the semimetallic MR above 5 T.

We further evaluate this model for the case of isotropic τ using the Fermi velocities of the ARPES-derived dispersion relation to obtain the mean-free-path (*35*). This additional evaluation tests the robustness of these conclusions under anisotropy in ℓ. In particular, where the FS hybridizes we find up to 2× suppression of the Fermi velocity, which may lead to a locally reduced ℓ. The variation of ℓ around the FS in this case as well as the magnetotransport results are shown in Fig. S11. Except for details at the lowest magnetic field due to a different mobility balance between the pockets, we observe almost identical results to the isotropic-ℓ model. This result shows that the suppression of v_F which naturally emerges where the FS reconstructs in the Peierls picture is by itself insufficient to create effective impedances to orbital motion. Above a few Tesla, the anisotropy of the FS quickly washes out, the MR saturates and QOs are expected.

The fourth and final iteration of this model is to implement effective impedances at the points where the FS reconstructs. Due to the complexity of the FS reconstruction, the only coherent way we found to approach this problem is by introducing hotspots wherever the FS folds back onto itself, including between pockets and bands. Impedances are again given a Gaussian shape as above, but with a fixed *k*-radius of 2×10^6 m^{-1} instead of an energy criterion which is no longer accessible. This results in many more impedances than shown in the main article, where only intrapocket hotspots are considered. The result is robust LMR as shown in Fig. S12 albeit with a slope that is 3 times too high. The Hall effect in this impeded version is back to hole-like despite the full FS reconstruction due to the effective suppression of turning points on the FS which dominate the Hall response. We thus suggest that the Peierls reconstruction takes place, but that effective impedances are only formed where ARPES shows a clear CDW gap. This led us to the model shown in the main article. At this time, unifying impeded cyclotron motion and the Peierls reconstruction is not possible phenomenologically and likely requires insight into the microscopic interactions which drive both phenomena.

In this section we have presented a detailed evaluation of the expected consequences of the Peierls reconstruction on the magnetotransport and QOs in $NbSe_2$. These calculations show that sharp FS corners and semimetallicity do leave a substantial mark on the MR, but that the results are not in agreement with experiment. In particular, without impedances, we expect the MR to change *H*-linear slope around 7 T, saturate above 20 T and for a myriad of QOs to appear below 30 T. These aspects are all incompatible with the experimental observations.

C. Beyond the RTA

The success of this simple impeded cyclotron motion model within the RTA to explain the observed MR and lack of QOs is noteworthy. With only the cold scattering rate as a single fit parameter it is able to account for many aspects of the experimental data, including the *H*-linear MR, the low turnover scale, the Kohler scaling, the low- and high- temperature MR, the qualitative behavior at the CDW transition and the lack of QOs in clean samples. This consistency with a wide range of experimental results with minimal degrees of freedom is our main result.

Nevertheless, the model remains phenomenological and has limitations. In this section we dive deeper into the following physical aspects; the non-collinear velocity between the endpoints of hotspots and the possibility of small-angle scattering particularly at intermediate temperatures (both explained below). In order to progress our understanding of the MR in $NbSe_2$ as much as possible, we here present additional state-of-the-art modeling beyond the RTA to explore these open questions. We emphasize, however, that in our experience thus far these more complex models fail to account for additional features in the experiment. Nevertheless, these computations serve to imply that small-angle scattering is not relevant to $NbSe_2$ and that the removal of spectral weight or another non-scattering origin is more likely than scattering off fluctuations as the source of the impedances to cyclotron motion.

One clear theoretical limitation of the RTA is non-parallel velocity between the endpoints of hotspots; i.e. $\vec{v}_{\vec{k}} + \vec{v}_{\vec{k}+\vec{Q}} \neq 0$. This is visualized in Fig. S13. Consider the scenario where the quasiparticle is quickly scattered back and forth between the two endpoints of a specific hotspot due to e.g. fluctuations or domain wall scattering. After a short time, this results in an even probability for the quasiparticle to maintain $\vec{v}_{\vec{k}}$ or $\vec{v}_{\vec{k}+\vec{Q}}$. In the RTA, the assumption is made that after this time no net contribution to the current remains, i.e. the average velocity vanishes or $\vec{v}_{\vec{k}} + \vec{v}_{\vec{k}+\vec{Q}} = 0$. In quasi-1D systems this assumption is reasonable given the predominantly anti-

parallel and similarly sized velocities on the right-moving and left-moving FS sheets. However, $NbSe_2$ is a quasi-2D material, however, and as such, strong $\vec{v}_{\vec{k}} + \vec{v}_{\vec{k}+\vec{Q}} \neq 0$ violations naturally occur. For the highlighted hotspot in Fig. S13, the velocity component indeed relaxes according to the fast or hot scattering rate along the black dashed direction, but a substantial component $\vec{v}_{\vec{k}} + \vec{v}_{\vec{k}+\vec{Q}} \neq 0$ remains in the direction of the gray dashed line which instead decays according to the much slower background cold scattering rate. The non-collinear velocity thus results in a rotation rather than complete decay of the net velocity and $\vec{l}$ and $\vec{v}$ are no longer aligned as assumed in the RTA. We thus obtain a bifurcation of the relaxation time for different velocity directions at fixed $\vec{k}$, which is beyond the (standard) RTA and few works exist which explore this regime.

This raises an important question about the nature of the hotspots: If the impedances are caused by a shift of spectral weight away from the Fermi level or otherwise terminate quasiparticle coherence, then it is appropriately modeled by impedances in the relaxation time as presented in the main article. However, if the impedances merely scatter charge back and forth elastically between two hotspots akin to a fluctuation regime usually found above T_{CDW}, then the non-collinear velocity at hotspots will result in further contributions to the conductivity. In Ref. (*23*,*47*,*67*) it was shown that exactly such non-collinear velocities at hotspots result in large corrections to the Hall effect and may even explain the sign change in electron-doped cuprates. The calculations in the RTA have implicitly assumed the first scenario by effectively impeding the charge at the impedance. Is impeded cyclotron motion robust under these latter effects?

In this section, we introduce a new Boltzmann framework to investigate the consequences of the latter hypothesis (scattering hotspot). This framework generalizes previous work aimed at investigating small-angle scattering, which we therefore also consider. We show that 1) scattering hotspots remain effective impedances to cyclotron motion but with increased turnover scale and reduced LMR, 2) the resulting MR is inconsistent with experiment, 3) that small-angle scattering maintains effective impedances with the same LMR but with a higher turnover scale and 4) small-angle scattering matches experiment less well than large-angle scattering.

D. Developing the Boltzmann transport framework

We have outlined above that the main issue to be investigated is the non-collinear velocity at hotspots. In Ref. (*67*) this was investigated using Kubo formalism in the low-field limit. Here, $\omega_c\tau$ is sufficiently high however that the problem is more difficult. After colliding with a hotspot, charge will evolve under cyclotron motion and a substantial probability remains for charge to reach another hotspot and cascade into a complex pattern. However, the underlying physics can be modeled semiclassically, which we develop and use to move past the low-field limit.

We develop a computational implementation of old ideas by Pippard, who investigated the effect of small-angle scattering on the MR of elemental Cu (*68*). The central idea is to abandon an absolute position of a quasiparticle on the FS, but instead to define a probability distribution across the FS where the quasiparticle resides. This probability distribution starts as a delta-function and evolves over time in the statistical average of ~10^{23} electrons. Under small-angle scattering the appropriate process is diffusion; for hotspots, we simply include scattering matrix elements beyond nearest neighbors of the appropriate magnitude. The framework is strictly a generalization of the SCTIF (*45*,*46*). This new framework was recently developed by the present authors to investigate intraband (small-angle) vs interband (Umklapp) scattering in quasi-1D conductors, which results in a bifurcation of the RTA (*48*). Here, we expand the framework from quasi-1D to 2D and beyond the low-field ($\omega_c\tau \leq 0.1$) regime.

To proceed, we define a probability distribution across the FS, which is taken to be 2D and therefore a line. No objection exists to define a surface-distribution for a 3D FS other than computational complexity.

$$\int_{FS} \mathrm{d}k P(k) = 1 \tag{S7}$$

where $P(k) \geq 0$ at all times. As in the RTA, we use the FS from Ref. (*35*). This probability distribution is not a superposition since phase information is neglected. $P(k) = |\Psi(k)|^2$ is more accurate since both represent quasiparticle probabilities, but we emphasize that P is considered in the statistical average of all electrons in the metal. For instance, elastic small-angle scattering from impurities results in Brownian motion of electrons across the FS (*68*). In its extreme small-angle limit, this process is modeled through diffusion of the probability distribution P across the FS, starting as a delta-function at zero time and turning into a homogeneous distribution at late times. This contrasts with the RTA in the SCTIF, in which charge is considered to no longer contribute at all to the conductivity after a scattering event and no preferential or structured scattering is considered. A key difference is also that in SCTIF at late times no charge remains, whereas in the new framework charge is strictly conserved and at late times the ensemble average velocity simply vanishes as $P(k)$ approaches thermal equilibrium. Eq. (S1) is rewritten as

$$\sigma_{ij} = \frac{e^2}{\pi\hbar} \int_{\mathrm{FS}} \frac{\mathrm{d}^2\vec{k}}{(2\pi)^2} \frac{v_i}{v} \int_0^\infty \mathrm{d}t \int_{FS} \mathrm{d}k' v_j(k') P(k', t) \tag{S8}$$

SCTIF is formally recovered by using $P(k',t) = \delta(k'-k(-t))\exp(\int_0^t \mathrm{d}t'/\tau(-t'))$. Thus, the norm of P (the total charge) exponentially vanishes as the charge is unaccounted for after scattering in the RTA. However, this removal of charge from the framework is no longer necessary. We may instead maintain strict charge conservation $Q = \int_{FS} \mathrm{d}k P(k) = 1$ at all times and diffuse the quasiparticle probability across the FS such that at late times $\langle \vec{v} \rangle = 0$, where the average represents integration of the probability over the FS $\langle \vec{v} \rangle = \int_{FS} \mathrm{d}k P(k) \vec{v}(k)$. This formalism neglects the phase of the wave function as well as any energetics of the dynamics. These two simplifications are linked, by neglecting the energetics of any dynamics, no phase differences can accrue from $\exp(-i\Delta\varepsilon t/\hbar)$ phases due to a quasiparticle spending a time t at energy $\Delta\varepsilon$ away from the Fermi level. In this manner, a wide variety of charge-conserving dynamics can be implemented in a semiclassical fashion beyond the RTA. This includes the small-angle scattering and hotspot scattering of interest here.

Using this extension of the SCTIF, we incorporate scattering dynamics beyond the RTA on $P(\vec{v},t)$.

$$\frac{\partial P}{\partial t} = D \frac{\partial^2 P}{\partial k^2} + \frac{1}{\tau_h} \sum_i \left(P(\vec{k} + \vec{Q}_i) - P\right) \delta\left(\varepsilon_{\vec{k}+\vec{Q}_i}\right) \tag{S9}$$

The first term is diffusion with coefficient D setting the cold scattering rate, the second term is hotspot scattering, which is only active where a Q-vector connects the FS. Where not shown, the time and k dependence of P are $P(\vec{k}, t)$ for clarity. The RTA of both terms is implemented as follows

$$\frac{\partial P}{\partial t} = -\frac{P}{\tau_c} - \frac{1}{\tau_h} \sum_i P\delta\left(\varepsilon_{\vec{k}+\vec{Q}_i}\right) \tag{S10}$$

In both approaches, the scattering matrix contains two terms. The first term is the cold scattering rate. In the RTA, this follows the exponential decay equivalent to large-angle scattering, while beyond the RTA, we follow Pippard (*68*) and implement small-angle scattering in its extreme limit, namely as a diffusion of the probability $P(k)$ over time. Large-angle scattering can be implemented as well in the new formulism, e.g. isotropic using $\frac{1}{\tau}\sum_i(P(k_i) - P)$. The second term is the hot scattering rate which only acts where the FS is connected by one of the Q-vectors. In the RTA this also follows exponential quasiparticle decay, while beyond the RTA we preserve charge and scatter between the endpoints of the hotspot. All scattering terms are implemented by discretizing the FS and creating a scattering matrix $\dot{P_i} = M_{ij}P_j$ of the momentary derivative. We then use Runge-Kutta 4 to evolve the probability distribution by a discrete timestep. The fundamental timesteps taken are typically 1/100 of the hot scattering time. In order to progress to the time where the velocity has fully relaxed (about 10× the cold scattering time), we must raise M to a power of over 10^9, which is achieved in about 10^4 (unequally spaced) steps in order to integrate over time. Between steps we evaluate up to M^{10^6} using binary exponentiation to save time on raising powers of the matrices.

Next, we implement cyclotron motion. In Ref. (*48*) both cyclotron motion and scattering were incorporated through the equations of motion. Over a given timestep, however, the discrete k-shift due to cyclotron motion and the k-distance between subsequent points on the discretized FS will not coincide. Nevertheless, in order to obtain the desired net rotation of the quasiparticle distribution P, one must split the probability into two proportions. For example, if the distance between two k-states is 1×10^7 m^{-1} yet the cyclotron motion over an individual timestep is 10^6 m^{-1}, then 10% of the total probability is transferred in the direction of cyclotron motion to the nearest neighbor. In the absence of any scattering, however, this partial transfer will result over time in a broadening of the probability distribution and introduce an artificial diffusion process limiting quasiparticles to about one rotation around the FS independent of the number of FS points; $\omega_c\tau \leq 2\pi$ (this artificial decay of $\langle v\rangle$ follows an exponential due to the central limit theorem). This artifact was not an issue in Ref. (*48*) since the primary results required $\omega_c\tau < 0.1$. For impeded cyclotron motion in $NbSe_2$, however, this is insufficient.

Here, we solve this problem and expand the validity of the model to arbitrary magnetic field strength. Ultimately, cyclotron motion is not a scattering or probabilistic event but rather a set rotation of all states. This is achieved by shifting the underlying basis, the discrete points on the FS themselves, at every time step. This change is non-trivial because the distance between subsequent k-positions shrinks or expands under cyclotron motion with the anisotropy of the effective mass. We therefore must renormalize the weights P_i such that the total probability for charge to be in a specific interval remains constant under cyclotron motion during each step and no charge is created or removed. At the same time, the movement of k-states requires re-computing the scattering matrix M during each evaluation, significantly increasing the computational cost. Finally, diffusion processes must be implemented in a stable fashion, which takes extra consideration due to the necessarily unequal distribution of the k points on the FS which is additionally changing as a function of time due to the small but finite anisotropy of the FS from Ref. (*35*). Details and the full implementation can be found in Ref. (*65*). The code is tested against Drude theory (including under magnetic field for Hall effect and MR) as well as impeded cyclotron motion and reproduces the results of the main article and Ref. (*21*) if Eq. (S10) is used to reproduce the RTA results.

In Fig. S14 we show the resulting MR in four different regimes. The first regime reproduces the RTA results of the main article within the new formalism. In this scenario, charge decays

exponentially for both cold and hot scattering, albeit with different timescales. The result is robust LMR. Next, we introduce small-angle scattering for the cold charge. Physically, this is relevant above the temperature where phonon scattering dominates over impurity scattering, while remaining well below the Debye temperature where phonons of sufficiently large q are thermally available to scatter all across the FS. Small-angle scattering is also relevant if the impurities are believed to reside between the 2D layers, as in cuprates (*69*). The result is again LMR of similar magnitude due to the continued existence of the hotspots, but with a prolonged quadratic regime at low field, in contrast with the experimental observation. We also note that with small-angle scattering the hot spots are no longer irrelevant at zero magnetic field, but reduce the zero-field lifetime by up to 5 times. We expect such a sizable effect to be observable as an upturn in $\rho_{ab}(T)$ while cooling through T_{CDW}, in contrast to experiment.

The third model explores the main challenge set out at the start of this section; i.e. to maintain the RTA for cold charge, but let charge continue with a 50% probability after reaching a hotspot – to account for the non-collinear velocity – and continued cyclotron motion thereafter. The resulting quasiparticle trajectories form a cascade or waterfall as repeated encounters with hotspots fragment the distribution $P(k)$. The resulting MR shows a significantly increased turnover scale and reduced H-linear slope compared to the RTA model. Conceptually, these results are analogous with an effective increase in the distance between hotspots.

The fourth and final model combines small-angle scattering with the new hotspot scattering. This calculation preserves charge at all times and relies on the convergence of the distribution P to equilibrium such that at late times $\langle v \rangle$ vanishes. The result is a quadratic MR over the entire field range available to experiment. At fields above 30 T, LMR does persist, showing that ICM is still valid although the response is shifted to considerably higher magnetic field.

These four models, computed using the newly developed framework, show that the type of scattering for both the cold and hot charge has important consequences for the MR. Reminiscent of the work of Pippard on elemental copper, we find that the presence of sharp k-space features exaggerates the differences between small- and large-angle scattering, albeit here due to hotspots instead of sharp FS corners. Secondly, we find that small-angle scattering results in a longer quadratic regime at low magnetic fields while barely affecting the high-field LMR regime. The large-angle scattering assumed in the RTA is closer to the low turnover scale observed experimentally than small-angle scattering. Thirdly, we find that non-collinear velocities at (fluctuation-driven) hotspots maintain LMR, but with a strongly reduced slope and an increased turnover scale compared to the RTA version. Finally, combining small-angle and hotspot scattering we have demonstrated a fully charge-conserving model with LMR, albeit at a field strength that is too high, proving nonetheless that ICM does not violate charge conservation. Among these models, the RTA version which assumes large-angle cold scattering rate (likely due to point-like impurities in the planes) and a termination of charge at impedances (e.g. through spectral weight shifted away from the Fermi level) matches best with experiment. We emphasize more parameter space exists to be explored beyond the RTA, and the strong influence of details of the scattering processes keeps the possibility open to find a unified description for the MR and Hall effect in $NbSe_2$.

Survey of other materials

Given that the microscopic origin of ICM is not obvious, we compare here our results to other CDW materials and again find varying results. A number of compounds show MR compatible with a Peierls-like FS reconstruction. The sister compound 1T-$TiSe_2$ shows both QOs

from the commensurate CDW reconstructed FS and a conventional MR (*70*,*71*). The commensurate CDW phase of 4*H*-TaS_2 also shows a saturating MR and QOs (*72*,*73*) and the same holds for 1*T*-$TaTe_2$ (*74*). The non-CDW compound 2*H*-NbS_2 shows a small quadratic MR with no signs of linearity, similar to what is observed in $NbSe_2$ outside the CDW phase (*75*). $NbSe_2$ is set apart among these dichalcogenides by its low T_{CDW}=33 K, the incommensurate nature of its CDW and the small fraction of density of states at the Fermi level affected by the CDW order.

In contrast to the above, a number of other compounds have shown behavior akin to our observations in $NbSe_2$. Thick films of VSe_2, for example, show a non-saturating *H*-linear MR with a slope of 0.11 $\mu\Omega$cm (of similar magnitude to that observed in $NbSe_2$) (*76*,*77*), though the disorder content in VSe_2 remains too high for any conclusion about QOs and possible saturation. In 2*H*-TaS_2 LMR was found which can be suppressed together with the commensurate CDW phase upon intercalation revealing QOs only outside the CDW phase despite the increase in disorder (*78*). A different report, however, shows QOs in the parent compound with unknown MR (*79*). The tritellurides $TbTe_3$ and $HoTe_3$ show LMR only inside the incommensurate CDW phase for which hotspots have been proposed as the origin (*80*,*81*), with studies extending to higher magnetic fields reporting magnetic breakdown and QOs (*82*). Finally, in $BaFe_2As_2$ not only the fluctuation regime near the QCP under doping, but also the spin density wave phase itself are also potential ICM candidates. Various groups have reported LMR which rapidly onsets just below T_{SDW} (*3*,*5*,*83*), while another study using cleaner crystals has revealed a large, quadratic MR as well as QOs (*84*). Empirically, therefore, a role for finite disorder in combination with density wave order to realize LMR appears plausible, yet more systematic studies are required.

Expectation of quantum oscillations in 2*H*-$NbSe_2$

The real-space QO radius can be estimated via the semiclassical equation $\ell_0 = \hbar k_F/Bq$. Neglecting the less than 10% variation in FS radius k_F across the Nb pockets, the cyclotron radius at 30 T is 75-120 nm for the six large, unreconstructed, Nb-derived pockets (see Table S1). In a FS reconstruction scenario for the CDW order, myriad QOs are expected with frequencies in the range 50-2000 Tesla, similar to what has been observed from 1 T in 4*H*-TaS_2, from 3 T in 2*H*-TaS_2 or to below 10 T in 2*H*-$TaSe_2$ (*72*,*79*). Such orbits have even smaller real-space radii down to 10 nm.

Comparing this characteristic scale to other measurements, we deduce that QOs are expected to be observed. Scanning tunneling microscopy studies have detected domains well above this size in clean $NbSe_2$ (*50*). The zero-field resistivity in our crystals is well described by a lifetime of 0.3 ps, which corresponds to a mean-free-path of 80 nm. While the mean-free-path relevant to QOs may, in principle, differ from this estimate, we remark here that the lifetime extracted from a Dingle analysis of the pancake pocket in the literature is in close agreement (*32*) and that $NbSe_2$ is neither strongly correlated nor a Dirac semimetal (scenarios where large differentials between the mean-free-path relevant to resistivity and QOs have been observed).

It is also instructive to compare with the observed QOs of the pancake pocket. Our measurements in Fig. S5 are to the best of our knowledge the first report of Shubnikov-de Haas oscillations in $NbSe_2$, i.e. measured in the resistivity, and are in good agreement with previous magnetization (de Haas-van Alphen) reports (*31*,*32*). Naturally, we cannot resolve QOs below the superconducting transition and strong oscillations emerge directly above H_{c2} where the radius is reduced to approximately 50 nm (though the shape of these orbits is highly anisotropic). In the

past, de Haas-van Alphen measurements have observed QOs below H_{c2} with a large characteristic real-space radius of 250 nm starting at only 2 T (*31*,*32*). Under these conditions, the cyclotron orbits must be formed despite the presence of superconducting vortices. Indeed, experimentally the Dingle temperature was found to be 40% higher in the mixed state than beyond (*31*), suggesting even larger orbits could be observed in the absence of superconductivity.

All these estimates and comparisons indicate that QOs from the reconstructed FS are expected to be observable, yet none have been reported and we also observe none here up to 30 T. It will be intriguing to explore whether QOs emerge finally once $NbSe_2$ is tuned beyond the CDW phase, e.g. through the application of pressure.

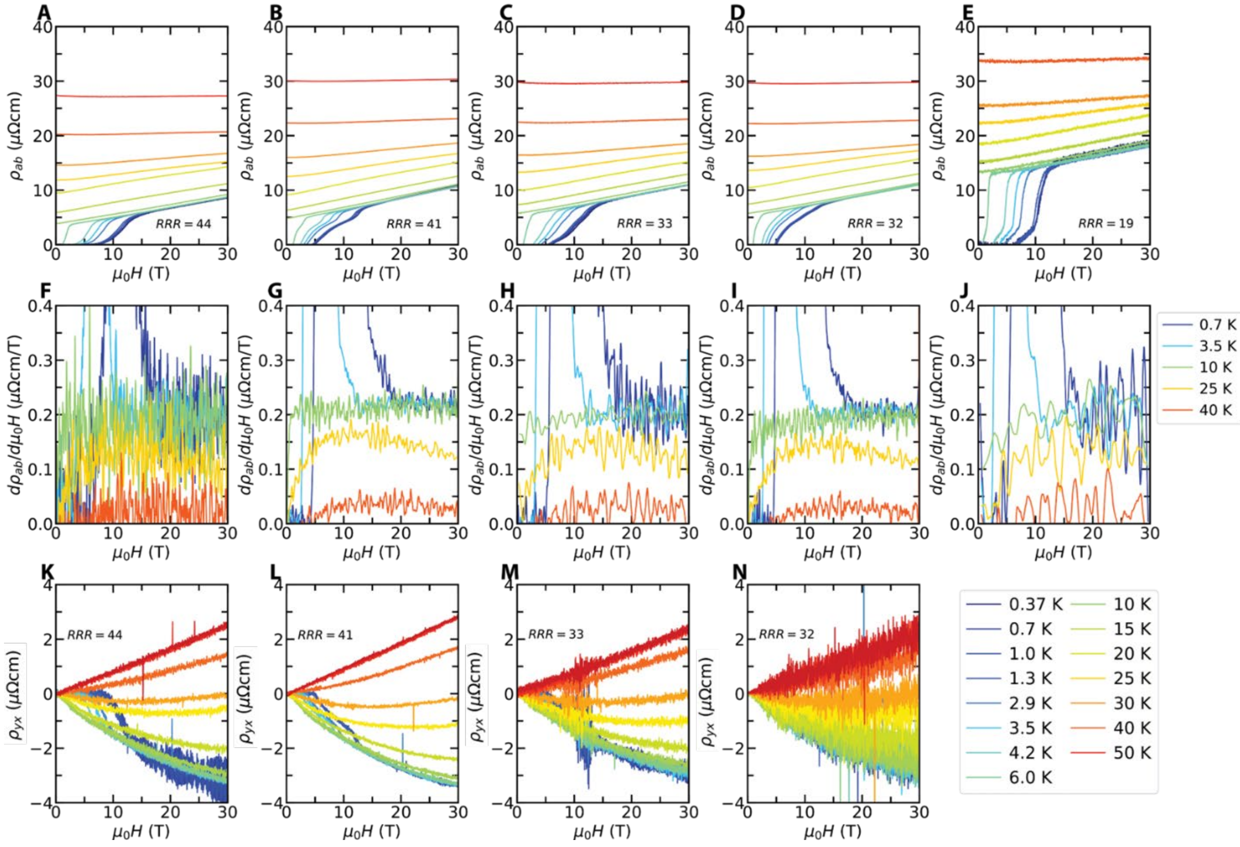

Fig. S1. Overview of the full high-field dataset. A)-E) Complete datasets of five samples with *RRR* values ranging from 19 to 44. All samples show the same qualitative behavior in their MR. Note how the zero field resistivity of all curves broadly increases with decreasing *RRR* to within experimental error. **F)-J)** Derivatives of the curves shown in panels A)-E) at selected temperatures. The curves in panels G)-J) have been smoothed using a Savitsky-Golay algorithm with polynomial order 1, while the curves shown in panel F) are raw data. **K)-N)** Hall resistivity $\rho_{yx}(H)$ measured on the samples with RRR = 44, 41, 33 and 32, respectively. (The Hall contact on the sample with RRR = 19 broke during cooldown.) Note that R_H ($=\rho_{yx}(H)/\mu_0 H$) changes sign at the temperature where the H-linearity of ρ_{yx} is lost. The oscillations on the low-T curves in panel I) are due to field-induced vibrations in the setup.

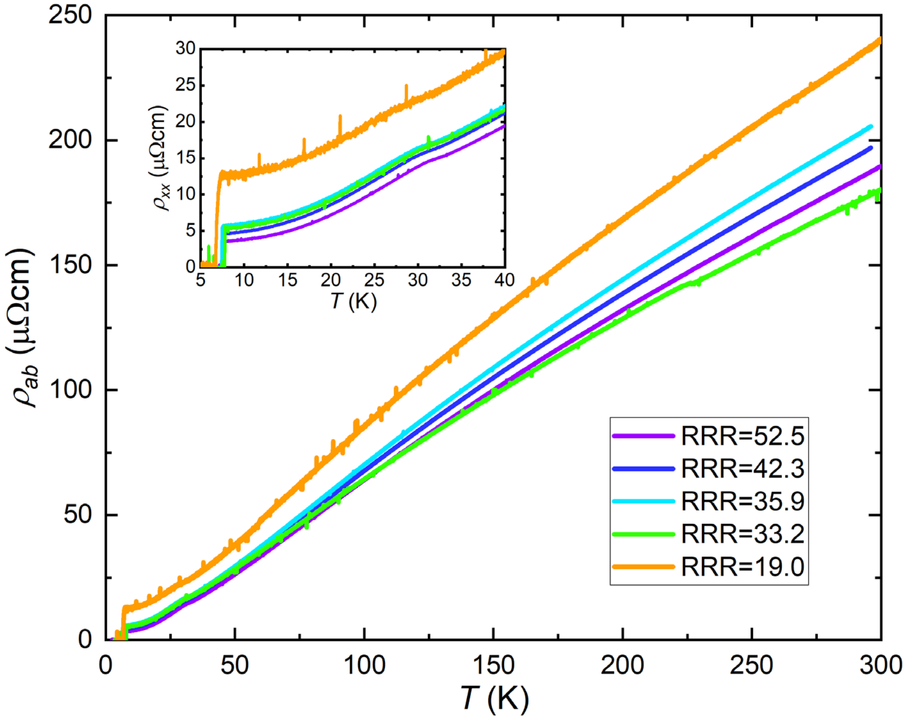

Fig. S2. $\rho_{ab}(T)$ curves of the $NbSe_2$ samples studied at low field. Cooldown curves of the five samples studied in low field in the CFMS. The samples with *RRR* = 52, 42 and 36 were cut from the same mother crystals as the high-field samples with *RRR* = 44, 41 and 33, respectively. The samples with *RRR* = 33 and *RRR* =19 are the same samples as the high-field samples with *RRR* = 33.2 and *RRR* = 19.4, but with fresh electrical contacts. We see that at high temperatures, Matthiessen's rule is violated, while below T_{CDW}, it is recovered. Inset: Zoom in at low *T* showing Matthiessen's rule is obeyed at low temperature.

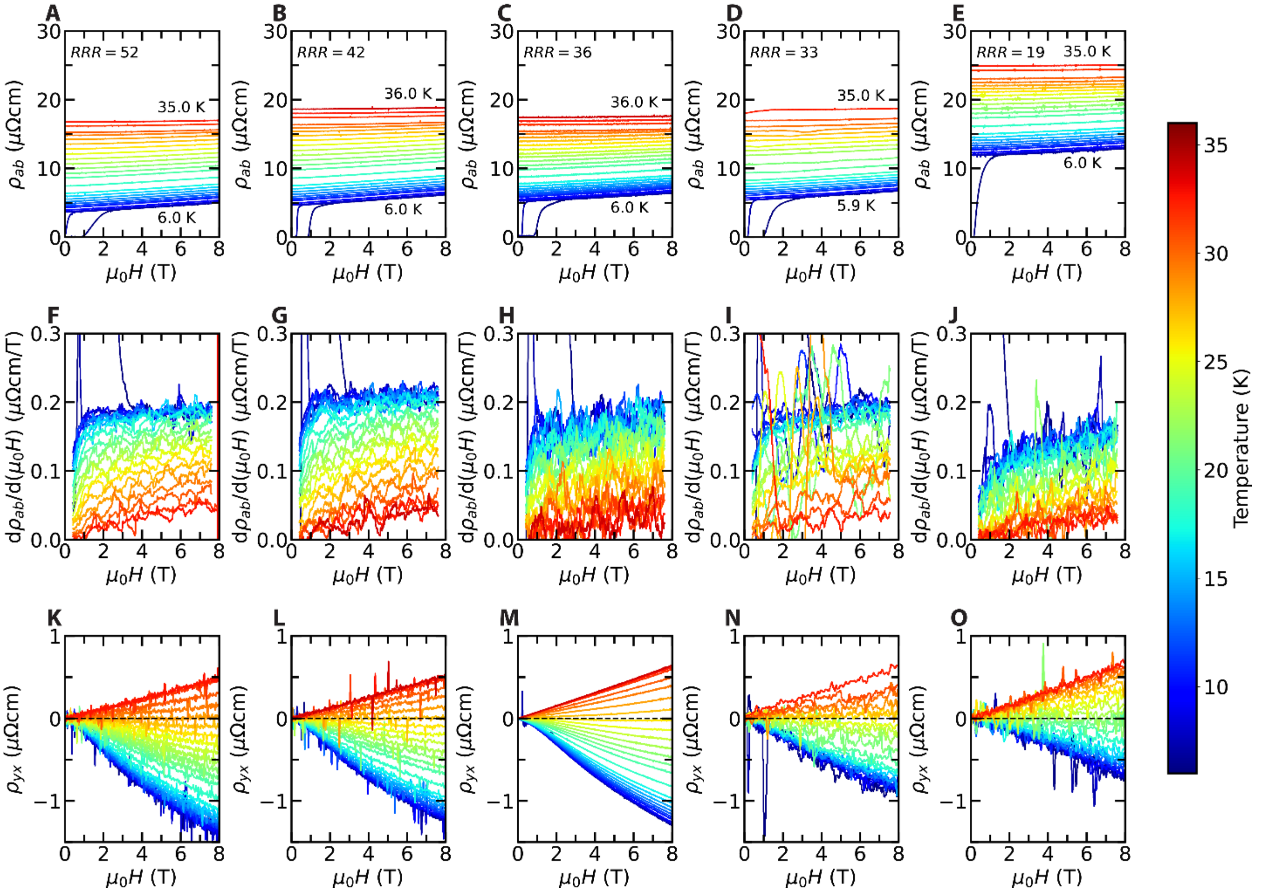

Fig. S3. Overview of the low-field data set. The samples with *RRR* = 52, 42 and 36 were cut from the same mother crystals as the high-field samples with *RRR* = 44, 41 and 33, respectively. The samples with *RRR* = 33 and *RRR* = 19 are the same samples as the high-field samples with *RRR* = 33.2 and *RRR* = 19.4, but with fresh electrical contacts. All curves have been measured in temperature steps of 1 K or 2 K. **A)-E)** ρ_{ab} as a function of field up to 8 T for the five measured samples. Note a gradual increase of the zero-field resistivity for increasing *RRR*. The minimum and maximum temperature at which a field sweep was performed have been indicated in the figure. **F)-J)** Derivatives of the curves shown in panels A)-E). These curves clearly show the breakdown of *H*-linearity for temperatures above 18 K. The fluctuations in I are due to equipment issues. **K)-O)** The Hall resistivity measured on the five crystals. The overall shape of the Hall effect is not too affected by disorder, but the magnitude at lower temperatures does seem to be affected. Note that the Hall effect changes sign, indicated by a near-zero ρ_{yx}, exactly at the temperature where *H*-linearity breaks down. The $\rho_{yx}(H)$ curves have been smoothed using a Gaussian filter.

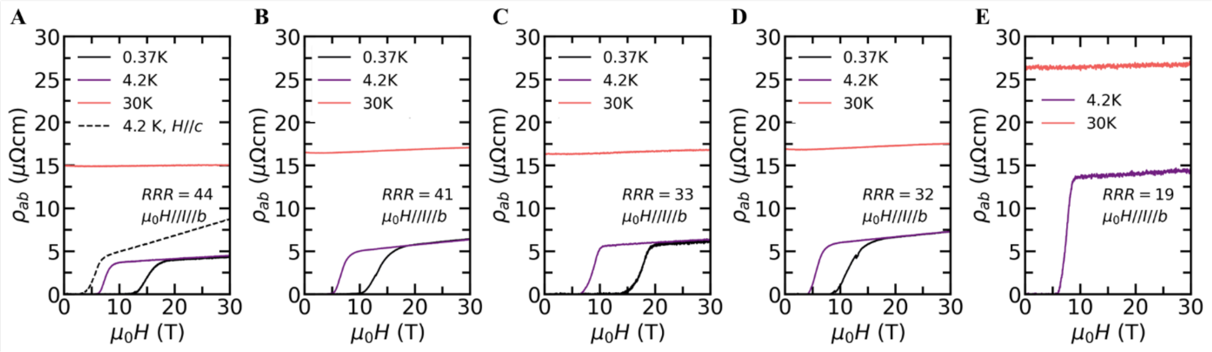

Fig. S4. Evidence for the orbital nature of the low-*T* MR in $NbSe_2$. A)-E) In-plane resistivity as a function of magnetic field for selected temperatures, with the field oriented parallel to the current. In this configuration, we observe a significant reduction in the magnitude of the MR, implying that the MR presented in the main article is predominantly of orbital character. This disfavors a non-orbital contribution to the MR as a mechanism for the observed MR profile. In panel A), the 4.2 K curve for $\vec{\boldsymbol{H}}//c$ has been included for comparison.

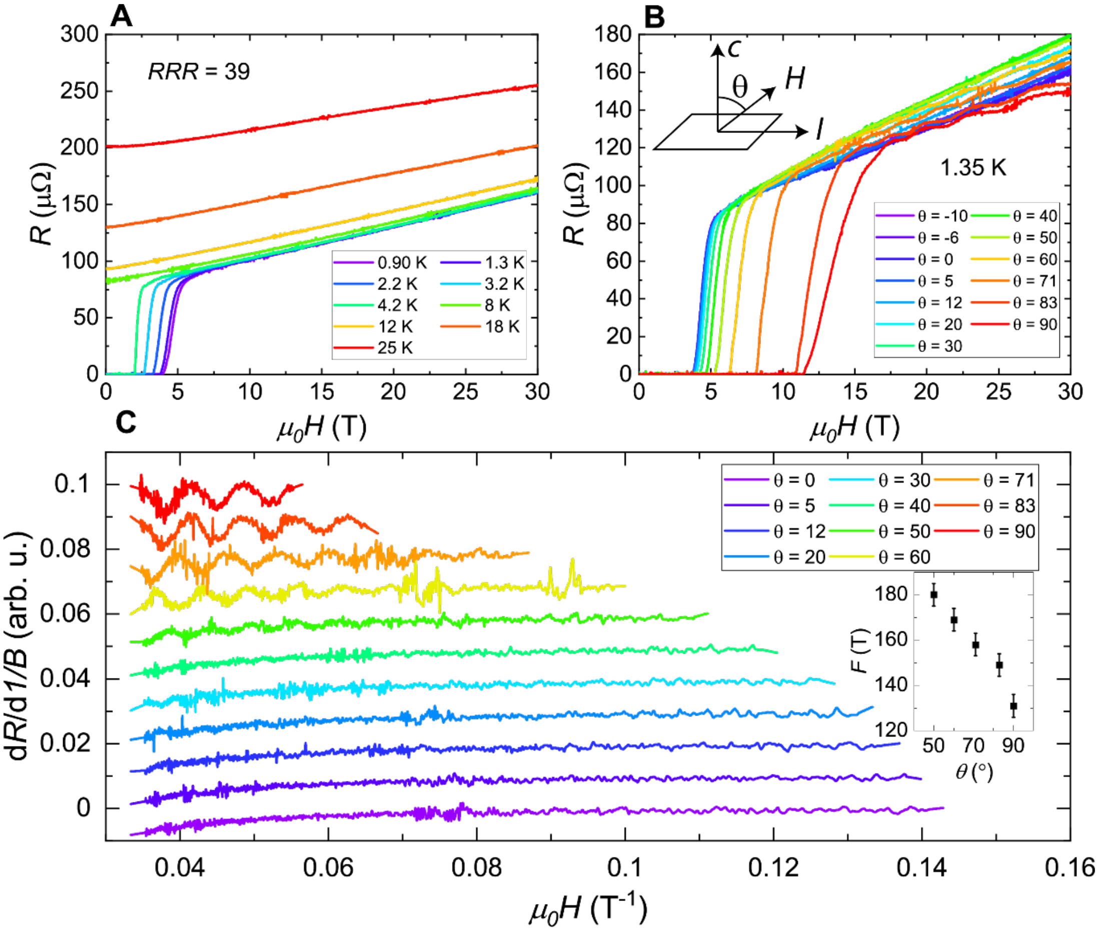

Fig. S5. Angle dependence of a sample with *RRR* = 39. A) Resistance R as a function of out-of-plane magnetic field. This sample was only measured in the temperature range where the MR is linear. **B**) Angle dependence of the resistance of $NbSe_2$ at $T = 1.35$ K. The angle θ is defined with respect to the c-axis, as shown in the inset. The MR remains linear up to θ=40°, although the slope gradually increases. For $\theta > 50°$, the MR quickly diminishes and becomes non-linear. At this angle, we also see an onset of QOs. **C)** Waterfall plot of the QOs. We observe no QOs for $\theta < 50°$. Beyond $\theta = 50°$, we observe QOs that increase in amplitude and decrease in frequency, suggesting that they originate from the pancake-like pocket at the Γ-point in the BZ as reported in Ref. (*32*). Curves have been offset for clarity. Inset shows the QO frequency as a function of θ. Note that the electrical contacts for this sample were mounted differently to the other crystals in this study. Here, contacts were attached onto the top surface in a Van der Pauw geometry, and the c-axis was not shorted. As a result, there is a contribution from $\boldsymbol{\rho_c}$ in this data set that likely contributes to the non-monotonic behaviour of the MR with respect to angle.

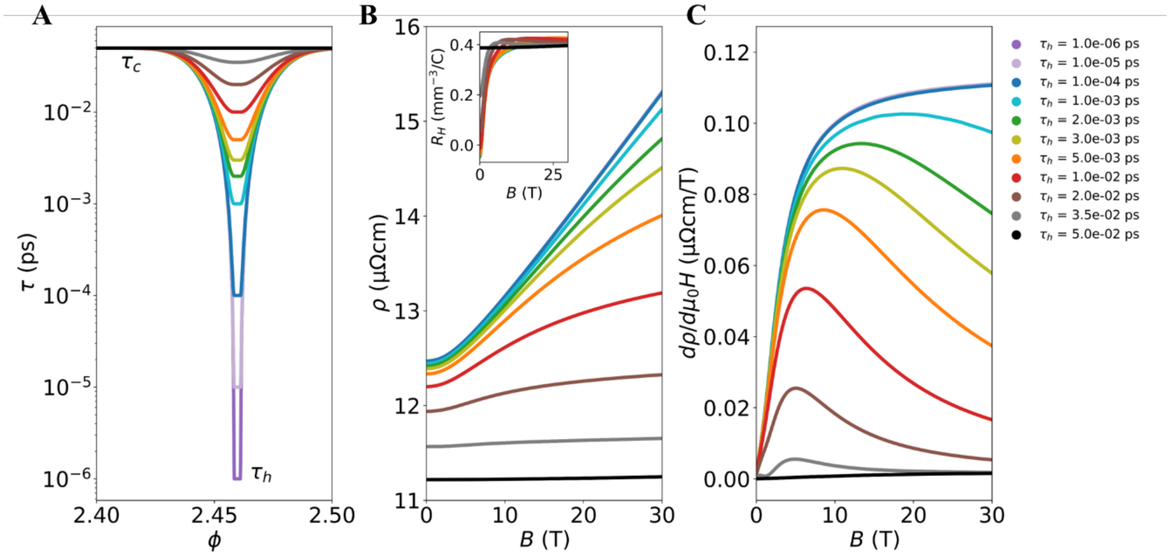

Fig. S6. Hot scattering rate dependence of the model and breakdown. A) Representative shape of a hotspot on the inner Γ pocket defined by Eq. (S3)-(S4) at fixed Δ = 10 meV and $\boldsymbol{\tau_c} =$ 0.05 ps for T = 25 K. The change in zero-field resistivity results from the fraction of the Fermi surface covered by hotspots. **B)** Resistivity as a function of hotspot strength. The inset shows that the hotspots leave the high-field Hall effect largely unchanged from the isotropic-τ result and is hole-like (black line). **C)** Corresponding derivative of the MR. Above a critical value, the hotspot strength is irrelevant in the fully formed ICM regime. As the hotspot is weakened, charge develops a finite probability for breakdown. This manifests in the MR through saturation which matches the shape and field range observed experimentally (see Fig. 3 of the main text or Fig. S1).

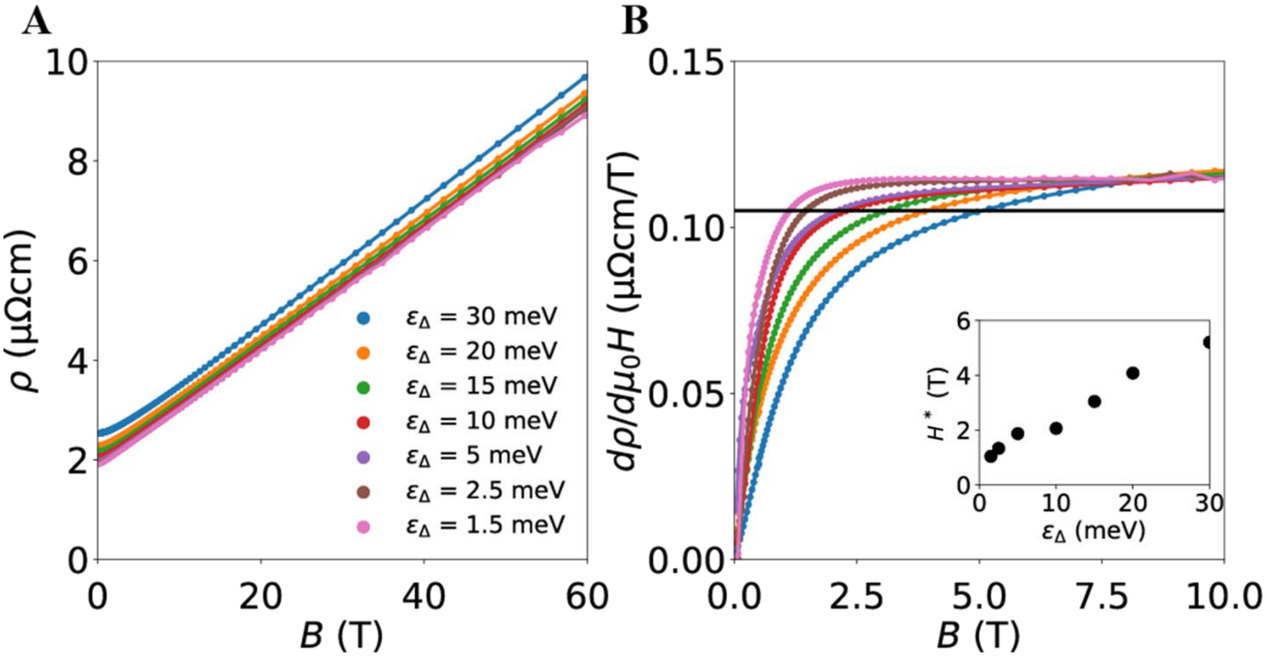

Fig. S7. Dependence of the model on ε_Δ. A) Resistivity as a function of Δ, where higher values correspond to wider hotspots. The change in zero-field resistivity is the consequence of the change in cold carrier density. **B)** Derivative of the data in panel A) in the low-field regime. The data show the *H*-linear MR is barely affected while the turnover scale increases with increasing ε_Δ. The inset shows quantitatively the evolution of H^*, where the MR reaches 90% of the high-field *H*-linear slope (indicated by the horizonal black line in the main panel). $\tau_c = 3$ ps is used to obtain a realistic zero-field resistivity for experiment at low *T*, while τ_h is kept sufficiently low that no magnetic breakdown is possible.

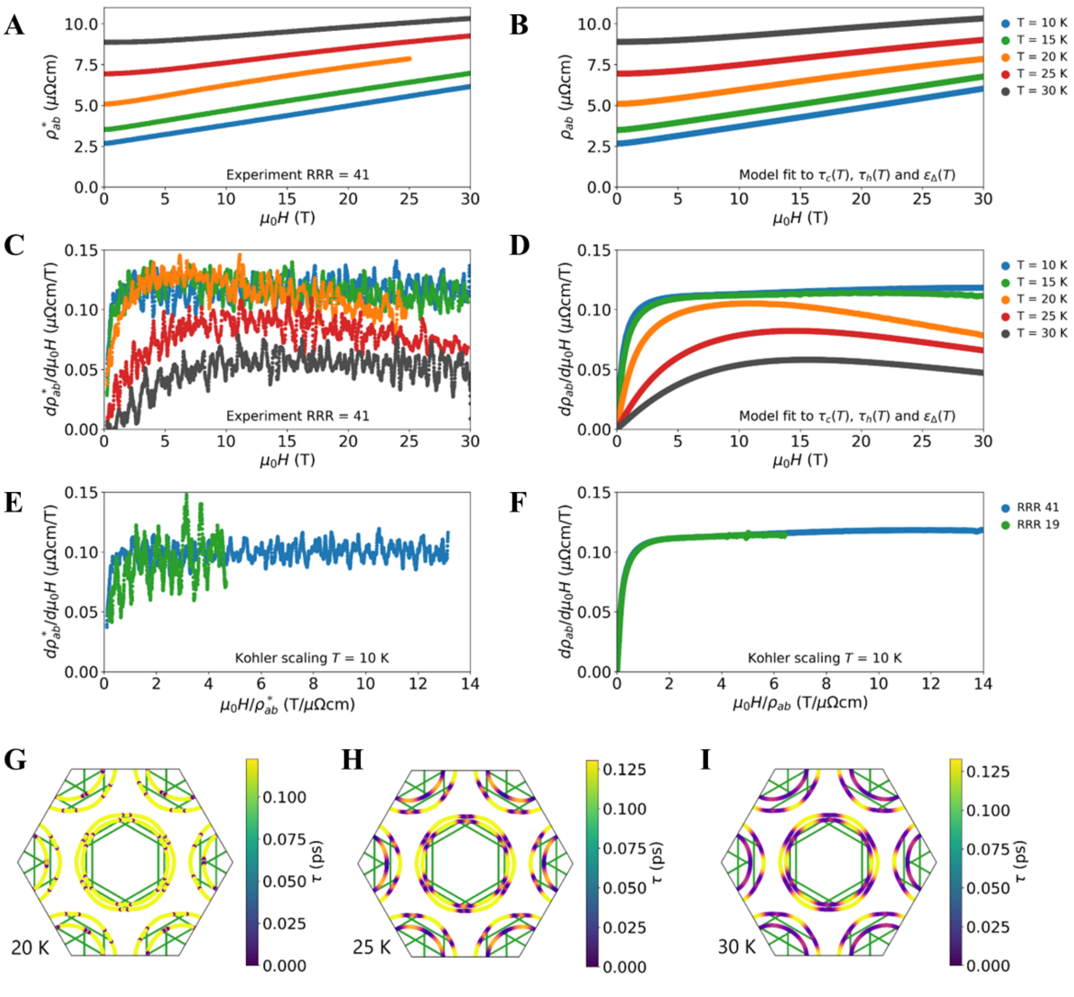

Fig. S8: Fit to the full (T, B, disorder) dependence of the MR of $NbSe_2$. A) Resistivity data for the *RRR* = 41 sample with the highest signal to noise. Note here that the experimental data have been renormalized by 40 % in order to match the room temperature resistivity value of 110 $\mu\Omega$cm reported in Ref. (*37*) and the LMR slope of the model. Note too that the 20 K sweep experienced temperature drift above 25 T and as a result, those data are not shown. **B)** Corresponding fit using all degrees of freedom in the model, i.e. $\boldsymbol{\tau_c}$, $\boldsymbol{\tau_h}$ and $\boldsymbol{\epsilon_\Delta}$. Values are listed in Table S2. **C)** Derivative of panel A). **D)** Derivative of panel B) showing a precise fit of not only the size but also the *H*-dependence of the MR in absolute units. **E)** Comparison with the most disordered sample at T = 10 K. **F)** The model nominally only changes $\boldsymbol{\tau_c}$ due to disorder and satisfies Kohler scaling as shown. If the CDW hotspots broaden with increased disorder, this would result in a higher turnover scale in the model, but such an effect could not be resolved experimentally. **G-I)** Scattering rate of the fitted model at temperatures where breakdown is observed and where the hotspot is neither arbitrarily strong nor wide. We find that the low $\boldsymbol{H^2}$ MR at elevated T can only be captured in the model by substantially broadening the hotspots on approach to T_{CDW}.

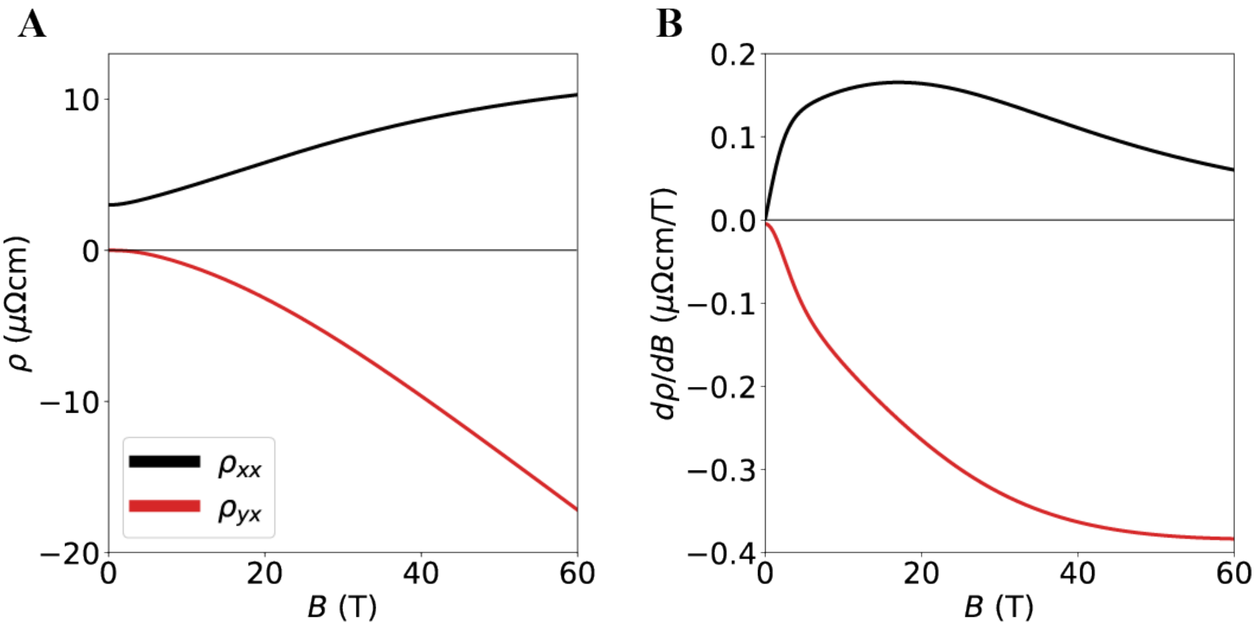

Fig. S9. MR and Hall resistivity post-reconstruction using Drude theory. A) $\rho_{xx}(H)$ and $\rho_{yx}(H)$ using the carrier density, effective mass and number of copies of each pocket in the BZ (see Table S1). The only degree of freedom is the (isotropic) scattering lifetime $\tau = 0.36$ ps corresponding to $\rho_{ab}(0) = 3\ \mu\Omega$cm. **B)** Corresponding derivatives. R_H can be obtained in mm^3/C by multiplying the red curve by 10. Notice that the Hall coefficient is negative yet vanishing at low field and double the size shown in the data in Fig. S1 at high fields. Meanwhile, the slope of the MR is of the right size, yet no extended *H*-linear regime exists and the quadratic MR at lowest magnetic field extends to over 5 T.

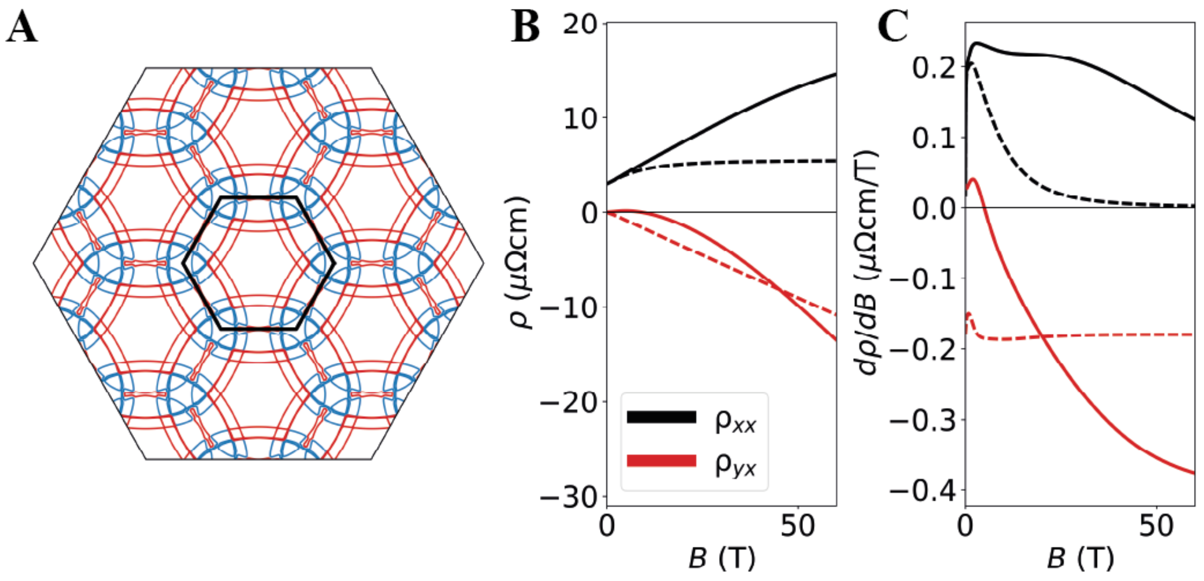

Fig. S10. Peierls scenario under isotropic-ℓ. A) Fermi surface after reconstruction. The outer hexagon is the normal-state BZ, the small hexagon the BZ in the CDW state neglecting the incommensurability. Blue lines represent hole-like pockets, red lines electron-like pockets. **B)** MR and Hall resistivity after matrix inversion obtained from Eq. (S6) using isotropic ℓ = 50 nm. The mean-free-path is the only degree of freedom and chosen to obtain $\rho_{ab}(0)$ = 3 $\mu\Omega$cm. Dashed lines represents the same MR calculation, but with all pockets rendered artificially electron-like, showing the large influence of the semi-metallic nature, especially at high field. **C)** Corresponding derivatives. Compared to Fig. S9 the low-field quadratic MR is strongly enhanced due to anisotropy of the FS, while the high-field MR is almost identical.

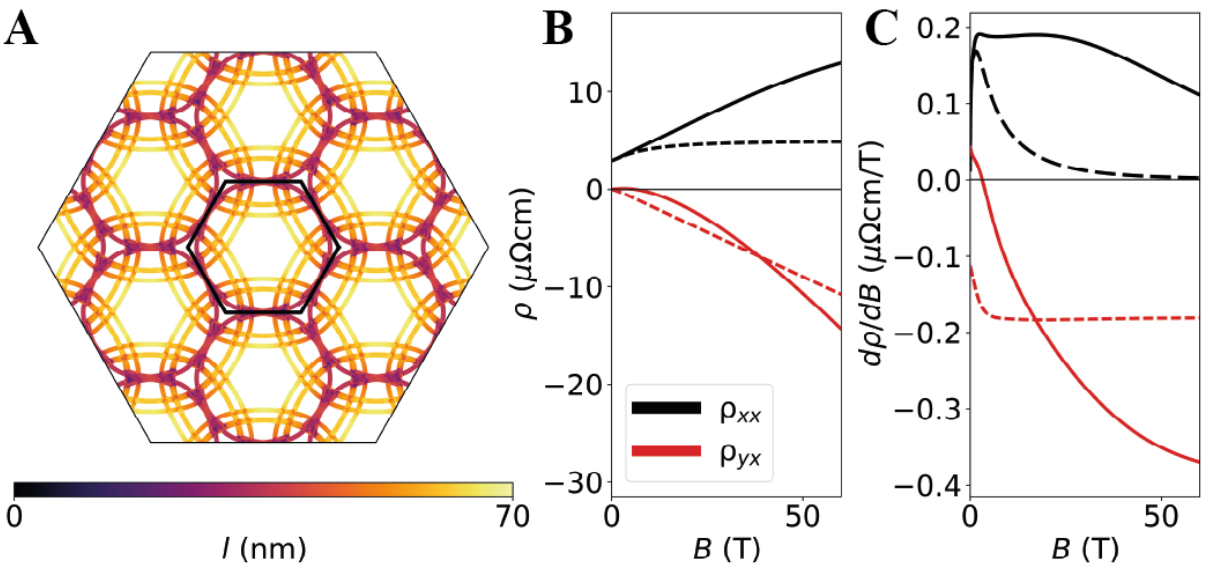

Fig. S11. Peierls scenario with isotropic τ. A) FS with the mean-free-path (Fermi velocity anisotropy) displayed in color. This anisotropy is obtained using $\ell = v_F\tau$ where isotropic $\tau = 0.25$ ps is the only degree of freedom chosen to match $\rho_{ab}(0) = 3$ μΩcm. **B)** Resistivity after matrix inversion obtained from Eq. (S6). Dashed lines represents the same MR calculation, but all pockets are artificially electron-like, showing the large influence of the semi-metallic nature, especially at high field. **C)** Corresponding derivatives. We obtain largely the same magnetotransport as shown in Fig S9.

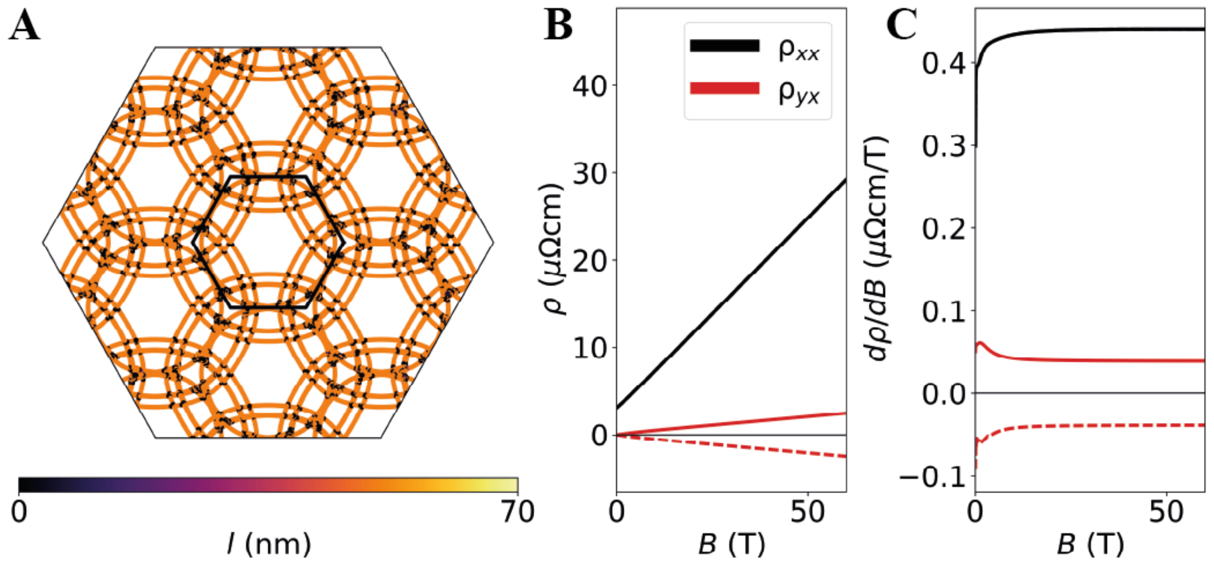

Fig. S12. Peierls scenario with impedances. A) FS with the mean-free-path displayed in color. The model is the same as in Fig. S10, except that where the FS hybridizes impedances are added. The zero-field resistivity rises by 3% due to the hotspots. **B)** Resistivity after matrix inversion obtained from Eq. (S1). Dashed line represents the same MR calculation, but with all pockets rendered artificially electron-like. Unlike above, the semi-metallicity is of minor importance to the MR and the Hall effect is hole-like, showing that impeded cyclotron motion dominates the magnetotransport. The semimetallic nature is largely irrelevant because $\omega_c\tau$ cannot grow larger than the inter-hotspot distance. **C)** Corresponding derivative. Unlike in Fig. S10, the MR is robustly linear to highest magnetic field.

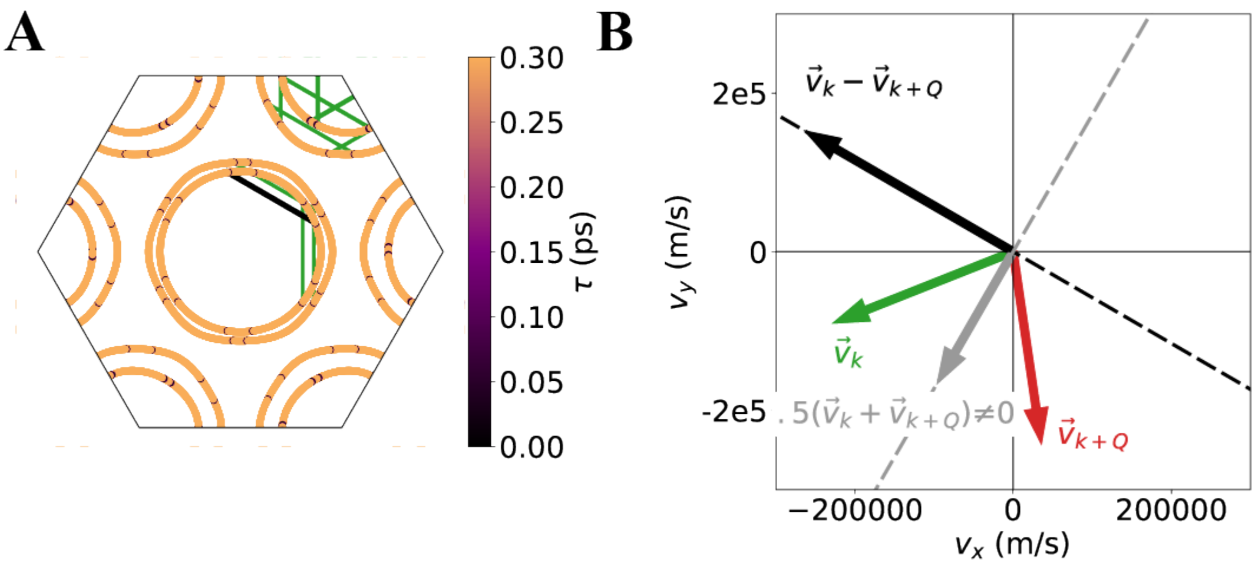

Fig. S13. Non-collinear velocity. A) Similar to Fig. 1B of the main article with one hotspot pair marked by the black line. The bottom right end of the black line is referred to as $\vec{\boldsymbol{v}}$ and top left is $\vec{\boldsymbol{v}}+\vec{\boldsymbol{Q}}$. **B)** The vector sum and difference of the corresponding velocities. At the hotspot, the black difference vector decays fast and the gray sum vector decays slowly. The latter is presumed to vanish in any RTA model. Consequently, a quasiparticle starting out as either green or red will have their net velocity direction quickly rotate to the gray direction and then slowly decay, resulting in a net current contribution which is no longer along the original velocity direction, i.e. $\vec{\boldsymbol{l}}$ cannot be written as $\vec{\boldsymbol{v}}\tau$ for any value of τ in violation of the RTA.

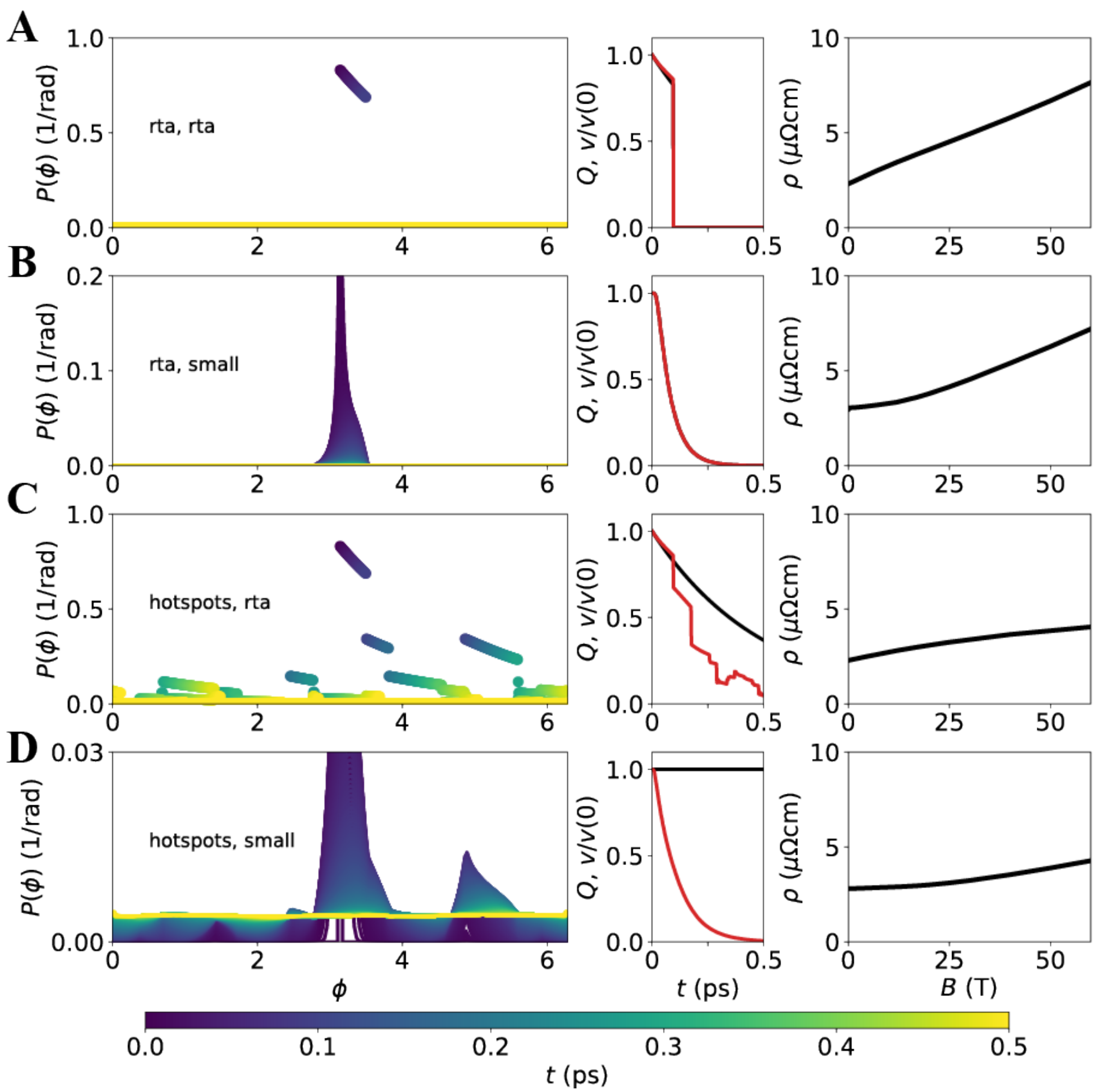

Fig. S14. Results beyond the RTA. A) Reproduction of the RTA results using the new formalism. The first panel shows the time evolution of $P(k)$ as a quasiparticle slowly decays until it reaches a hotspot and is impeded. The second column shows the total charge in the system (black) and the ensemble average velocity (red) over time. The third column shows the MR when the hot rate is sufficiently high to impede charge and the cold scattering rate matches the experimentally observed zero-field resistivity. **B)** Using small-angle scattering for the cold charge, the quasiparticle peak diffuses between nearby hotspots where charge terminates. The LMR persists but shows an extended quadratic regime at low magnetic fields. **C)** Using charge-conserving scattering between the ends of hotspots while keeping the RTA for cold scattering. The result is a complex cascade of repeated bifurcations in $P(k)$ whenever a hotspot is encountered. LMR remains, but with reduced slope and only above 25 T. **D)** Fully charge-conserving small-angle and hotspot scattering. LMR with reduced slope and increased turnover scale is obtained.

f (T)	mass (m_0)	LK damping	type	Carrier density n (10^{26} m^{-3})	# copies	location	$B_{QO,l}$ (T)	$B_{QO,\tau}$ (T)	R_c at 30 T
3907	2.49	0.978	hole	15.1	2	1 K		30	75
7490	2.28	0.981	hole	29.0	1	1 Γ		58	104
7604	2.42	0.979	hole	29.4	2	2 K		45	105
9522	2.74	0.973	hole	36.9	1	2 Γ		69	117
48	0.30	1.000	hole	0.185	6	K^*	4.8	5.8	8.3
315	0.71	0.998	hole	1.215	2	K^*	12.5	14.3	21
639	1.51	0.992	hole	2.462	2	K^*	25.4	29.6	30
41	0.68	0.998	electron	0.157	3	M^*	8.5	13.5	7.7
1079	0.86	0.997	electron	4.161	1	Γ	21.2	16.8	40
1706	1.26	0.994	electron	6.575	1	Γ	28.1	24.3	50
1907	1.24	0.994	electron	7.350	1	Γ	28.3	23.8	53
1957	1.28	0.994	electron	7.545	1	Γ	29.0	25.0	53

Table S1. Quantitative information per pocket. The top section refers to the unreconstructed FS (*35*), the bottom section to the reconstructed FS shown in Fig. S10A. Here, *c*-axis warping and the pancake pocket are neglected. The QO frequency f assumes $\vec{\boldsymbol{H}}//c$ determined via Onsager's relation $f = \hbar A_k/2\pi e$ where A_k is the cross-sectional area of the pocket. The effective cyclotron mass is determined via $1/m^* = \frac{\hbar^2}{2\pi}\frac{\mathrm{d}A_k}{\mathrm{d}\varepsilon}$ at the Fermi level. m_0 is the vacuum electron mass. LK stands for Lifshitz-Kosevich and this column shows the size of the QOs compared to zero temperature expected from damping at 30 T and 0.3 K. We conclude that thermal damping is not the reason why QOs are not observed. Carrier type is determined by whether the cross section of the orbit grows or shrinks with increasing energy. The carrier density is listed per copy but includes spin degeneracy via $A_k/2\pi^2 c$. The location column lists in the unreconstructed case the band number from the tight binding model (*35*) and the high symmetry point around or nearest to which the orbit is located. Stars (*) refer to the high symmetry location in the reconstructed BZ (where $\Gamma^* = \Gamma$). B_{QO} is the onset magnetic field where QOs are expected. We define $\omega_c\tau = 2\pi$ as the magnetic field at which charge has a probability $1/e \sim 0.37$ to complete an orbit. QOs are expected when $\omega_c\tau = 1$ while matching $\rho_{ab} = 2.5$ $\mu\Omega$cm at 0 T is estimated as the residual resistivity. The first estimate assumes the unimpeded isotropic-ℓ scenario of Fig S10 (ℓ = 60 nm), the second estimate assumes the unimpeded isotropic-τ scenario of Fig. S11 τ =3.0 ps). Note that experimentally we exceed both estimates of B_{QO} for all eight reconstructed Fermi pockets yet observe no QOs. $R_c = \hbar\bar{\boldsymbol{k}}/Be$ is the average real-space cyclotron radius, where $\bar{\boldsymbol{k}} = \sqrt{\boldsymbol{A_k}/\boldsymbol{\pi}}$ approximates the cross section of the orbit by a circle (independent of scattering model).

	10 K	15 K	20 K	25 K	30 K	10 K (Fig. 3)	25 K (Fig. 3)
τ_c (ps)	0.223	0.170	0.122	0.130	0.133	0.198	0.0575
τ_h (ps)	≤0.0002	~0.00035	0.0015	0.01	0.02	≤0.0002	0.00689
ϵ_Δ (meV)	~5	~5	10	50	100	20	20
$\rho_{\text{model}}(0)$ ($\mu\Omega$cm)	2.66	3.49	5.09	6.94	8.89	3.88	11.83
$\rho^*_{\text{exp}}(0)$ ($\mu\Omega$cm)	2.67	3.52	5.09	6.93	8.88	3.90	11.83

Table S2: Parameters used in Fig. S8 (to fit data for the *RRR* = 41 sample). The model is entirely defined by $\boldsymbol{\tau_c}$, $\boldsymbol{\tau_h}$ and $\boldsymbol{\epsilon_\Delta}$. $\boldsymbol{\epsilon_\Delta}$ controls the hotspot width and is used to fit the low-field $\boldsymbol{H^2}$ coefficient of the MR (see Fig. S7); $\boldsymbol{\tau_h}$ is used to fit the breakdown field where $\mathbf{d}\boldsymbol{\rho}/\mathbf{d}\boldsymbol{\mu_0 H}$ is at a maximum, and $\boldsymbol{\tau_c}$ is fitted to $\boldsymbol{\rho(0)}$ as listed. None of these parameters can be used to meaningfully modify the LMR slope itself. At the lowest temperatures, no breakdown is observed and the hotspots themselves are not fitted. Instead, a guess is taken for $\boldsymbol{\epsilon_\Delta}$ based on the high-T data. $\boldsymbol{\tau_h}$ is subsequently sufficiently small that no breakdown occurs in the accessible field range. As stated in the main text, at low T the model has only one meaningful degree of freedom, namely $\boldsymbol{\tau_c}$. Taking $\langle \boldsymbol{v} \rangle \approx \mathbf{2} \times \mathbf{10^5}$ m/s, the Mott-Ioffe-Regel limit may be defined as the lifetime ($\approx$ 0.0015 ps) where the mean-free-path equals $\boldsymbol{a}$, the unit cell parameter. The temperatures with meaningful data relating to $\boldsymbol{\tau_h}$ satisfy this limit. The lowest T results can be tuned above this limit by broadening the hot spots. The last two columns give the parameters used for the fits in Fig. 3 of the main article.

REFERENCES

1. C. Herring, Effect of random inhomogeneities on electrical and galvanomagnetic measurements. *J. Appl. Phys.* **31**, 1939–1953 (1960).

2. T. Khouri, U. Zeitler, C. Reichl, W. Wegscheider, N. E. Hussey, S. Wiedmann, J. C. Maan, Linear magnetoresistance in a quasifree two-dimensional electron gas in an ultrahigh mobility GaAs quantum well. *Phys. Rev. Lett.* **117**, 256601 (2016).

3. K. K. Huynh, Y. Tanabe, K. Tanigaki, Both electron and hole Dirac cone states in $Ba(FeAs)_2$ confirmed by magnetoresistance. *Phys. Rev. Lett.* **106**, 217004 (2011).

4. I. M. Hayes, R. D. McDonald, N. P. Breznay, T. Helm, P. J. W. Moll, M. Wartenbe, A. Shekhter, J. G. Analytis, Scaling between magnetic field and temperature in the high-temperature superconductor $BaFe_2(As_{1-x}P_x)_2$. *Nat. Phys.* **12**, 916–919 (2016).

5. N. Maksimovic, I. M. Hayes, V. Nagarajan, J. G. Analytis, A. E. Koshelev, J. Singleton, Y. Lee, T. Schenkel, Magnetoresistance scaling and the origin of *H*-linear resistivity in $BaFe_2(As_{1-x}P_x)_2$. *Phys. Rev. X* **10**, 041062 (2020).

6. S. Licciardello, N. Maksimovic, J. Ayres, J. Buhot, M. Čulo, B. Bryant, S. Kasahara, Y. Matsuda, T. Shibauchi, V. Nagarajan, J. G. Analytis, N. E. Hussey, Coexistence of orbital and quantum critical magnetoresistance in $FeSe_{1-x}S_x$. *Phys. Rev. Res.* **1**, 023011 (2019).

7. P. Giraldo-Gallo, J. A. Galvis, Z. Stegen, K. A. Modic, F. F. Balakirev, J. B. Betts, X. Lian, C. Moir, S. C. Riggs, J. Wu, A. T. Bollinger, X. He, I. Božović, B. J. Ramshaw, R. D. McDonald, G. S. Boebinger, A. Shekhter, Scale-invariant magnetoresistance in a cuprate superconductor. *Science* **361**, 479–481 (2018).

8. T. Sarkar, P. R. Mandal, N. R. Poniatowski, M. K. Chan, R. L. Greene, Correlation between scale-invariant normal-state resistivity and superconductivity in an electron-doped cuprate. *Sci. Adv.* **5**, eaav6753 (2019).

9. J. Ayres, M. Berben, M. Čulo, Y.-T. Hsu, E. van Heumen, Y. Huang, J. Zaanen, T. Kondo, T. Takeuchi, J. R. Cooper, C. Putzke, S. Friedemann, A. Carrington, N. E. Hussey, Incoherent transport across the strange-metal regime of overdoped cuprates. *Nature* **595**, 661–666 (2021).

10. M. Parish, P. Littlewood, Non-saturating magnetoresistance in heavily disordered semiconductors. *Nature* **426**, 162–165 (2003).

11. Z. H. Wang, L. Yang, X. J. Li, X. T. Zhao, H. L. Wang, Z. D. Zhang, X. P. A. Gao, Granularity controlled nonsaturating linear magnetoresistance in topological insulator Bi_2Te_3 films. *Nano Lett.* **14**, 6510–6514 (2014).

12. A. A. Abrikosov, Quantum magnetoresistance. *Phys. Rev. B* **58**, 2788–2794 (1998).

13. A. B. Pippard, *Magnetoresistance in metals*, Cambridge Studies in Low Temperature Physics (Cambridge Univ. Press, 1989).

14. A. E. Koshelev, Linear magnetoconductivity in multiband spin-density-wave metals with nonideal nesting. *Phys. Rev. B* **88**, 060412 (2013).

15. Y. Feng, Y. Wang, D. M. Silevitch, J.-Q. Yanc, R. Kobayashi, M. Hedo, T. Nakama, Y. Onuki, A. V. Suslov, B. Mihaila, P. B. Littlewood, T. F. Rosenbaum, Linear magnetoresistance in the low-field limit in density-wave materials. *Proc. Natl. Acad. Sci. U.S.A.* **116**, 11201–11206 (2019).

16. J. Ayres, M. Berben, C. Duffy, R. D. H. Hinlopen, Y.-T. Hsu, A. Cuoghi, M. Leroux, I. Gilmutdinov, M. Massoudzadegan, D. Vignolles, Y. Huang, T. Kondo, T. Takeuchi, S. Friedemann, A. Carrington, C. Proust, N. E. Hussey, Universal correlation between *H*-linear magnetoresistance and *T*-linear resistivity in high-temperature superconductors. *Nat. Commun.* **15**, 8406 (2024).

17. C. Boyd, P. W. Phillips, Single-parameter scaling in the magnetoresistance of optimally doped $La_{2-}xSrxCuO_4$. *Phys. Rev. B* **100**, 155139 (2019).

18. A. A. Patel, J. McGreevy, D. P. Arovas, S. Sachdev, Magnetotransport in a model of a disordered strange metal. *Phys. Rev. X* **8**, 021049 (2018).

19. J. Singleton, Temperature scaling behavior of the linear magnetoresistance observed in high-temperature superconductors. *Phys. Rev. Mater.* **4**, 061801 (2020).

20. A. E. Koshelev, Magnetotransport of multiple-band nearly antiferromagnetic metals due to hot-spot scattering. *Phys. Rev. B* **94**, 125154 (2016).

21. R. D. H. Hinlopen, F. A. Hinlopen, J. Ayres, N. E. Hussey, $\boldsymbol{B}^2$ to $\boldsymbol{B}$-linear magnetoresistance due to impeded orbital motion. *Phys. Rev. Res.* **4**, 033195 (2022).

22. J. Kim, E. Altman, S. Chatterjee, Linear magnetoresistance from glassy orders. *Proc. Natl. Acad. Sci. U.S.A.* **121**, e2405720121 (2024).

23. C. M. Duffy, S. J. Tu, Q. H. Chen, J. S. Zhang, A. Cuoghi, R. D. H. Hinlopen, T. Sarkar, R. L. Greene, K. Jin, N. E. Hussey, Evidence for spin-fluctuation-mediated superconductivity in electron-doped cuprates. arXiv:2502.13612 [cond-mat.supr-con] (2025).

24. G. Grissonnanche, Y. Fang, A. Legros, S. Verret, F. Laliberté, C. Collignon, J. Zhou, D. Graf, P. A. Goddard, L. Taillefer, B. J. Ramshaw, Linear-in temperature resistivity from an isotropic Planckian scattering rate. *Nature* **595**, 667–672 (2021).

25. M. R. Norman, H. Ding, M. Randeria, J. C. Campuzano, T. Yokoya, T. Takeuchik, T. Takahashi, T. Mochiku, K. Kadowaki, P. Guptasarma, D. G. Hinks, Destruction of the Fermi surface in underdoped high-T_c superconductors. *Nature* **392**, 157–160 (1998).

26. R. C. Morris, R. V. Coleman, R. Bhandari, Superconductivity and magnetoresistance in $NbSe_2$. *Phys. Rev. B* **5**, 895–901 (1972).

27. M. Naito, S. Tanaka, Galvanomagnetic effects in the charge-density-wave state of $2H$-$NbSe_2$ and $2H$-$TaSe_2$. *J. Phys. Soc. Jpn.* **51**, 228–236 (1982).

28. L. M. Falicov, P. R. Sievert, Theory of the galvanomagnetic effects in metals with magnetic breakdown: Semiclassical approach. *Phys. Rev.* **138**, A88–A98 (1965).

29. A. B. Pippard, Quantization of coupled orbits in metals: II. The two-dimensional network, with special reference to the properties of zinc. *Philos. Trans. A Math Phys. Eng. Sci.* **256**, 317–355 (1964).

30. A. B. Pippard, Magnetic breakdown in a dislocated lattice. *Proc. A* **287**, 165–182 (1965).

31. J. E. Graebner, M. Robbins, Fermi-surface measurements in normal and superconducting 2*H*-$NbSe_2$. *Phys. Rev. Lett.* **36**, 422–425 (1976).

32. R. Corcoran, P. Meeson, Y. Onuki, P.-A. Probst, M. Springford, K. Takita, H. Harima, G. Y. Guo, B. L. Gyorffy, Quantum oscillations in the mixed state of the type II superconductor 2*H*-$NbSe_2$. *J. Phys. Condens. Matter* **6**, 4479–4492 (1994).

33. Z.-Y. Cao, K. Zhang, A. F. Goncharov, X.-J. Yang, Z.-A. Xu, X.-J. Chen, Pressure effect of the charge density wave transition on Raman spectra and transport properties of 2*H*-$NbSe_2$. *Phys. Rev. B* **107**, 245125 (2023).

34. M. Kohler, Zur Magnetischen Widerstandsänderung Reiner Metalle. *Ann. Phys.* **424**, 211–218 (1938).

35. D. J. Rahn, S. Hellmann, M. Kalläne, C. Sohrt, T. K. Kim, L. Kipp, K. Rossnagel, Gaps and kinks in the electronic structure of the superconductor 2*H*-$NbSe_2$ from angle-resolved photoemission at 1 K. *Phys. Rev. B* **85**, 224532 (2012).

36. L.-J. Li, Z.-A. Xu, J.-Q. Shen, L.-M. Qiu, Z.-H. Gan, The effect of a charge-density wave transition on the transport properties of 2*H*-$NbSe_2$. *J. Phys. Condens. Matter* **17**, 493–498 (2005).

37. K. Cho, M. Kończykowski, S. Teknowijoyo, M. A. Tanatar, J. Guss, P. B. Gartin, J. M. Wilde, A. Kreyssig, R. J. McQueeney, A. I. Goldman, V. Mishra, P. J. Hirschfeld, R. Prozorov, Using controlled disorder to probe the interplay between charge order and superconductivity in $NbSe_2$. *Nat. Commun.* **9**, 2796 (2018).

38. W. Li, S. Pyon, A. Ichinose, S. Okayasu, T. Tamegai, Suppression of superconductivity in heavy-ion irradiated 2*H*-$NbSe_2$ caused by negative pressure. *J. Phys. Soc. Jpn.* **91**, 074709 (2022).

39. W. Li, S. Pyon, A. Yagi, T. Ren, M. Suyama, J. Wang, T. Matsumae, Y. Kobayashi, A. Takahashi, D. Miyawaki, T. Tamegai, Effects of 3 MeV proton irradiation on superconductivity and CDW in 2*H*- $NbSe_2$ single crystals. *J. Phys. Soc. Jpn.* **92**, 064701 (2023).

40. F. Flicker, J. van Wezel, Charge order from orbital-dependent coupling evidenced by $NbSe_2$. *Nat. Commun.* **6**, 7034 (2015).

41. F. Flicker, J. van Wezel, Charge order in $NbSe_2$. *Phys. Rev. B* **94**, 235135 (2016).

42. Z. Wang, C. Chen, J. Mo, J. Zhou, K. P. Loh, Y. P. Feng, Decisive role of electron-phonon coupling for phonon and electron instabilities in transition metal dichalcogenides. *Phys. Rev. Res.* **5**, 013218 (2023).

43. D. J. Huntley, R. F. Frindt, Transport properties of $NbSe_2$. *Can. J. Phys.* **52**, 74–117 (1974).

44. S. V. Borisenko, A. A. Kordyuk, V. B. Zabolotnyy, D. S. Inosov, D. Evtushinsky, B. Büchner, A. N. Yaresko, A. Varykhalov, R. Follath, W. Eberhardt, L. Patthey, H. Berger, Two energy gaps and Fermi-surface "arcs" in $NbSe_2$. *Phys. Rev. Lett.* **102**, 166402 (2009).

45. W. Shockley, Effect of magnetic fields on conduction—"Tube integrals". *Phys. Rev.* **79**, 191–192 (1950).

46. R. G. Chambers, The kinetic formulation of conduction problems. *Proc. Phys. Soc. A* **65**, 458–459 (1952).

47. H. Kontani, K. Kanki, K. Ueda, Hall effect and resistivity in high-T_c superconductors: The conserving approximation. *Phys. Rev. B* **59**, 14723–14739 (1999).

48. P. Chudzinski, M. Berben, X. Xu, N. Wakeham, B. Bernáth, C. Duffy, R. D. H. Hinlopen, Y.-T. Hsu, S. Wiedmann, P. Tinnemans, R. Jin, M. Greenblatt, N. E. Hussey, Emergent symmetry in a low dimensional superconductor on the edge of Mottness. *Science* **382**, 792–796 (2023).

49. N. Ramakrishnan, Y. T. Lai, S. Lara, M. M. Parish, S. Adam, Equivalence of effective medium and random resistor network models for disorder-induced unsaturating linear magnetoresistance. *Phys. Rev. B* **96**, 224203 (2017).

50. K. Iwaya, T. Hanaguri, A. Koizumi, K. Takaki, A. Maeda, K. Kitazawa, Electronic state of $NbSe_2$ investigated by STM/STS. *Phys. B Condens. Matter* **329-333**, 1598–1599 (2003).

51. N. V. Kozlova, N. Mori, O. Makarovsky, L. Eaves, Q. D. Zhuang, A. Krier, A. Patanè, Linear magnetoresistance due to multiple-electron scattering by low-mobility islands in an inhomogeneous conductor. *Nat. Commun.* **3**, 1097 (2012).

52. R. Peierls, Zur Theorie der elektrischen und thermischen Leitfähigkeit von Metallen. *Ann. Phys.* **396**, 121–148 (1930).

53. W. Kohn, Image of the Fermi surface in the vibration spectrum of a metal. *Phys. Rev. Lett.* **2**, 393–394 (1959).

54. M. D. Johannes, I. I. Mazin, Fermi surface nesting and the origin of charge density waves in metals. *Phys. Rev. B* **77**, 165135 (2008).

55. M. D. Johannes, I. I. Mazin, C. A. Howells, Fermi-surface nesting and the origin of the charge-density wave in $NbSe_2$. *Phys. Rev. B* **73**, 205102 (2006).

56. F. Weber, S. Rosenkranz, J.-P. Castellan, R. Osborn, R. Hott, R. Heid, K.-P. Bohnen, T. Egami, A. H. Said, D. Reznik, Extended phonon collapse and the origin of the charge-density wave in $2H$-$NbSe_2$. *Phys. Rev. Lett.* **107**, 107403 (2011).

57. T. Valla, A. V. Fedorov, P. D. Johnson, P.-A. Glans, C. McGuinness, K. E. Smith, E. Y. Andrei, H. Berger, Quasiparticle spectra, charge density waves, superconductivity, and electron-phonon coupling in $2H$-$NbSe_2$. *Phys. Rev. Lett.* **92**, 086401 (2004).

58. W. L. McMillan, Microscopic model of charge-density waves in 2*H*-$TaSe_2$. *Phys. Rev. B* **16**, 643–650 (1977).

59. C. M. Varma, A. L. Simons, Strong-coupling theory of charge-density-wave transitions. *Phys. Rev. Lett.* **51**, 138–141 (1983).

60. W. L. McMillan, Theory of discommensurations and the commensurate-incommensurate charge-density-wave phase transition. *Phys. Rev. B* **14**, 1496–1502 (1976).

61. A. Soumyanarayanan, M. M. Yee, Y. He, J. van Wezel, D. J. Rahn, K. Rossnagel, E. W. Hudson, M. R. Norman, J. E. Hoffman, Quantum phase transition from triangular to stripe charge order in $NbSe_2$. *Proc. Natl. Acad. Sci. U.S.A.* **110**, 1623–1627 (2013).

62. N. Doiron-Leyraud, C. Proust, D. LeBoeuf, J. Levallois, J.-B. Bonnemaison, R. Liang, D. A. Bonn, W. N. Hardy, L. Taillefer, Quantum oscillations and the Fermi surface in an underdoped high-T_c superconductor. *Nature* **447**, 565–568 (2007).

63. W. Tabis, Y. Li, M. Le Tacon, L. Braicovich, A. Kreyssig, M. Minola, G. Dellea, E. Weschke, M. J. Veit, M. Ramazanoglu, A. I. Goldman, T. Schmit, G. Ghiringhelli, N. Barišić, M. K. Chan, C. J. Dorow, G. Yu, X. Zhao, B. Keimer, M. Greven, Charge order and its connection with Fermi-liquid charge transport in a pristine high-T_c cuprate. *Nat. Commun.* **5**, 5875 (2014).

64. K. Momma, F. Izumi, *VESTA3* for three-dimensional visualization of crystal, volumetric and morphology data. *J. Appl. Crystal.* **44**, 1272–1276 (2011).

65. Data repository, https://doi.org/10.34973/drd0-kj23.

66. N. P. Ong, Geometric interpretation of the weak-field Hall conductivity in two-dimensional metals with arbitrary Fermi surface. *Phys. Rev. B* **43**, 193–201 (1991).

67. H. Kontani, Optical conductivity and Hall coefficient in high-T_c superconductors: Significant role of current vertex corrections. *J. Phys. Soc. Jpn.* **75**, 013703 (2006).

68. A. B. Pippard, The influence of small-angle scattering on metallic conduction. *Proc. A* **305**, 291–318 (1968).

69. C. M. Varma, E. Abrahams, Effective Lorentz force due to small-angle impurity scattering: Magnetotransport in high-T_c superconductors. *Phys. Rev. Lett.* **86**, 4652–4655 (2001).

70. P. Knowles, B. Yang, T. Muramatsu, O. Moulding, J. Buhot, C. J. Sayers, E. Da Como, S. Friedemann, Fermi surface reconstruction and electron dynamics at the charge-density-wave transition in $TiSe_2$. *Phys. Rev. Lett.* **124**, 167602 (2020).

71. R. D. H. Hinlopen, O. N. Moulding, W. R. Broad, J. Buhot, F. Bangma, A. McCollam, J. Ayres, C. J. Sayers, E. Da Como, F. Flicker, J. van Wezel, S. Friedemann, Lifshitz transition enabling superconducting dome around a charge-order critical point. *Sci. Adv.* **10**, eadl3921 (2024).

72. R. M. Fleming, R. V. Coleman, Oscillatory magnetotransport in the layer compounds 4H_b-TaS_2 and 2H-$TaSe_2$. *Phys. Rev. B* **16**, 302–315 (1977).

73. J. J. Gao, J. G. Si, X. Luo, J. Yan, Z. Z. Jiang, W. Wang, Y. Y. Han, P. Tong, W. H. Song, X. B. Zhu, Q. J. Li, W. J. Lu, Y. P. Sun, Origin of the large magnetoresistance in the candidate chiral superconductor 4H_b-TaS_2. *Phys. Rev. B* **102**, 075138 (2020).

74. H. Chen, Z. Li, L. Guo, X. Chen, Anisotropic magneto-transport and magnetic properties of low-temperature phase of $TaTe_2$. *EPL* **117**, 27009 (2017).

75. M. Naito, S. Tanaka, Electrical transport properties in 2H-NbS_2, -$NbSe_2$, -TaS_2 and -$TaSe_2$. *J. Phys. Soc. Jpn.* **51**, 219–227 (1982).

76. H. Liu, L. Bao, Z. Zhou, B. Che, R. Zhang, C. Bian, R. Ma, L. Wu, H. Yang, J. Li, C. Gu, C.-M. Shen, S. Du, H.-J. Gao, Quasi-2D transport and weak antilocalization effect in few-layered VSe_2. *Nano Lett.* **19**, 4551–4559 (2019).

77. Y. Xue, Y. Zhang, H. Wang, S. Lin, Y. Li, J.-Y. Dai, S. P. Lau, Thickness-dependent magnetotransport properties in 1T-VSe_2 single crystals prepared by chemical vapor deposition. *Nanotechnology* **31**, 145712 (2020).

78. W. Biberacher, A. Lerf, Electrical transport properties of 2*H*-TaS_2 intercalation compounds with variable charge transfer. *Mol. Cryst. Liq. Cryst.* **121**, 149–152 (1985).

79. S. J. Hillenius, R. V. Coleman, Quantum oscillations and Fermi surface of 2*H*-TaS_2. *Phys. Rev. B* **18**, 3790–3798 (1978).

80. A. A. Sinchenko, P. D. Grigoriev, P. Lejay, P. Monceau, Linear magnetoresistance in the charge density wave state of quasi-two-dimensional rare-earth tritellurides. *Phys. Rev. B* **96**, 245129 (2017).

81. A. Fang, N. Ru, I. R. Fisher, A. Kapitulnik, STM studies of $TbTe_3$: Evidence for a fully incommensurate charge density wave. *Phys. Rev. Lett.* **99**, 046401 (2007).

82. P. Walmsley, S. Aeschlimann, J. A. W. Straquadine, P. Giraldo-Gallo, S. C. Riggs, M. K. Chan, R. D. McDonald, I. R. Fisher, Magnetic breakdown and charge density wave formation: A quantum oscillation study of the rare-earth tritellurides. *Phys. Rev. B* **102**, 045150 (2020).

83. R. Kumar, S. Singh, S. Nair, High temperature linear magnetoresistance and scaling behavior in the $Ba(Fe_{1-x}Co_x)_2As_2$ series. arXiv:1801.03768v1 [cond-mat.supr-con] (2018).

84. T. Terashima, N. Kurita, M. Kimata, M. Tomita, S. Tsuchiya, H. Satsukawa, A. Harada, K. Hazama, M. Imai, A. Sato, S. Uji, K. Kihou, C.-H. Lee, H. Kito, Y. Tomioka, T. Ito, A. Iyo, H. Eisaki, T. Liang, M. Nakajima, S. Ishida, S.-i. Uchida, T. Saito, H. Fukazawa, Y. Kohori, H. Harima, Quantum oscillations in iron-based superconductors: $BaFe_2As_2$ vs. KFe_2As_2. *J. Phys. Conf. Ser.* **449**, 012022 (2013).
